# Genetic Dissection of Femoral and Tibial Microarchitecture

**DOI:** 10.1002/jbm4.10241

**Published:** 2019-11-11

**Authors:** Lu Lu, Jinsong Huang, Fuyi Xu, Zhousheng Xiao, Jing Wang, Bing Zhang, Nicolae Valentin David, Danny Arends, Weikuan Gu, Cheryl Ackert‐Bicknell, Olivia L Sabik, Charles R Farber, Leigh Darryl Quarles, Robert W Williams

**Affiliations:** ^1^ Department of Genetics, Genomics and Informatics University of Tennessee Health Science Center Memphis TN USA; ^2^ Department of Medicine University of Tennessee Health Science Center Memphis TN USA; ^3^ Department of Molecular and Human Genetics Baylor College of Medicine Houston TX USA; ^4^ Department of Medicine Northwestern University Feinberg School of Medicine Chicago IL USA; ^5^ Breeding Biology and Molecular Animal Breeding Humboldt University Berlin Germany; ^6^ Department of Orthopaedic Surgery and Biomedical Engineering University of Tennessee Health Science Center Memphis TN USA; ^7^ Center for Musculoskeletal Research University of Rochester Rochester NY USA; ^8^ Center for Public Health Genomics University of Virginia Charlottesville VA USA

**Keywords:** ANIMAL MODEL; CORTICAL BONE; GENE ONTOLOGY; GENOME‐WIDE ASSOCIATION STUDIES; IGNOROME; μCT; QUANTITATIVE TRAIT LOCUS; SEX DIFFERENCE; SYSTEMS GENETICS; TRABECULAR BONE

## Abstract

Our understanding of the genetic control of bone strength has relied mainly on estimates of bone mineral density. Here we have mapped genetic factors that influence femoral and tibial microarchitecture using high‐resolution x‐ray computed tomography (8‐μm isotropic voxels) across a family of 61 BXD strains of mice, roughly 10 isogenic cases per strain and balanced by sex. We computed heritabilities for 25 cortical and trabecular traits. Males and females have well‐matched heritabilities, ranging from 0.25 to 0.75. We mapped 16 genetic loci most of which were detected only in females. There is also a bias in favor of loci that control cortical rather than trabecular bone. To evaluate candidate genes, we combined well‐established gene ontologies with bone transcriptome data to compute bone‐enrichment scores for all protein‐coding genes. We aligned candidates with those of human genome‐wide association studies. A subset of 50 strong candidates fell into three categories: (1) experimentally validated genes already known to modulate bone function (*Adamts4, Ddr2*, *Darc*, *Adam12, Fkbp10*, *E2f6*, *Adam17, Grem2, Ifi204*); (2) candidates without any experimentally validated function in bone (eg, *Greb1*, *Ifi202b*), but linked to skeletal phenotypes in human cohorts; and (3) candidates that have high bone‐enrichment scores, but for which there is not yet any functional link to bone biology or skeletal system disease (including *Ifi202b, Ly9, Ifi205, Mgmt, F2rl1, Iqgap2*). Our results highlight contrasting genetic architecture between sexes and among major bone compartments. The alignment of murine and human data facilitates function analysis and should prove of value for preclinical testing of molecular control of bone structure. © 2019 The Authors. *JBMR Plus* published by Wiley Periodicals, Inc. on behalf of American Society for Bone and Mineral Research.

## Introduction

The development and maintenance of the skeletal system is modulated by thousands of genetic variants, as well as many environmental factors. In massive genome‐wide association studies (GWASs), variation in stature, for instance, has been linked securely to over 3000 DNA variants.^1^, ^2^ Similarly, over the past decade more than 1000 loci and gene variants have been defined in human, mouse, and rat cohorts that control BMD, risk of fracture, and morphometric traits.^3^ One limitation is that most large genetic studies of osteoporosis have exploited DXA to quantify areal BMD.^4^ In addition, a subset of studies have exploited pQCT to define subsets of variants that modulate bone volumetric BMD (vBMD).^5^, ^6^ Although BMD accounts for about 70% of bone strength, this measurement does not provide the 3D structural precision of high‐resolution μCT.^7^, ^8^, ^9^, ^10^ Three‐dimensional maps of structure and architecture generated by μCT have many advantages, including (1) minimal interference from intra‐ and extraosseous soft tissues, (2) high‐content data acquisition, and (3) isotropic resolution as high as 6 μm.^11^, ^12^ Finally, the development of finite element analysis of μCT data makes it possible to model mechanical properties of bone.^13^

Experimental rodent models can be used efficiently to evaluate candidate genes discovered in GWASs and convert variants to mechanisms and potentially even to treatments.^14^ Although the use of knockouts and knockins of single mutations is a well‐established approach to test gene function, an alternative unbiased approach is to map quantitative trait loci (QTL) that influence natural variation in bone structure using large families of genetically diverse animals.^15^ In this way, the clinical range and complexity of bone disease can be captured, while retaining tight control over diet, environment, and genotypes. For example, the mouse diversity panel^16^, ^17^ and the BXD family have been used to define and confirm roles for *Asxl2* in BMD and *Alpl* in hypophosphatasia.^18^ In comparison to intercrosses, these large families of isogenic, but diverse strains can be used to systematically test gene‐by‐environmental interactions, to establish replicability, and to test new therapies and treatments.^19^ Families of strains, such as the BXDs, are also advantageous because deep genomic, metabolic, metagenomic, and phenotypic data have already been generated.^20^, ^21^ It therefore becomes practical to compare variation in bone and the skeletal system with those of many other traits in many other systems, and at multiple levels of organization.^18^, ^22^, ^23^, ^24^, ^25^

In this study, we have combined deep quantitative phenotyping of bone microstructure with a fine‐grained genetic dissection to understand the control of bone architecture, focusing on femur and tibia. We have systematically evaluated cortical and trabecular compartments.

We have also developed and applied a method to rank essentially all protein‐coding genes in mammals with respect to their potential roles in bone biology. We define a new set of genes that have a strong likelihood of being related to bone biology, but that currently lack any relevant literature. We refer to this set as a “bone ignorome.^26^, ^27^ We have merged information on the ignorome with our set of candidate genes to rank those most likely to contribute to variation in bone structure and function.

## Materials and Methods

### Animals

All experimental procedures were in accordance with the *Guidelines for the Care and Use of Laboratory Animals* published by the National Institutes of Health and were approved by the Animal Care and Use Committee at the University of Tennessee Health Science Center (UTHSC; Memphis, TN, USA).

The BXD family was housed in a single specific pathogen‐free (SPF) facility at UTHSC, and maintained at approximately 22°C on a 14/10 hours light/dark cycle. Cases were provided Agway Prolab 3000 (5% fat; Agway, Syracuse, NY, USA) chow and Memphis aquifer tap water *ad libitum*. Sixty‐one BXD strains and both parental strains, C57BL/6J (B6) and DBA/2 J (D2), were sacrificed for tissue harvest. Cases ranged in age from 50 to 375 days with an average of 100 days. A total of 597 animals were studied: 290 females and 307 males. A total of 576 femurs and 515 tibias were harvested. Differences between numbers of animals and bones reflect breakage or loss. Precise numbers of strains vary by trait, sex, and age, but all parameters were evaluated using 50 to 63 unique strains. For details on sample sizes, see Supplementary Data S1 and data in http://GeneNetwork.org. Many cases analyzed here had been previously studied by Zhang and colleagues,^28^ although here we have integrated additional data, including 239 new cases and 15 additional strains. After dissection and removal of soft tissue, femurs and tibias were stored in 75% ethanol until measurement.

### μCT measurements

High‐resolution x‐ray computed tomography (μCT40, Scanco Medical, Basserdorf, Switzerland) was used to scan and measure morphometric parameters of femurs and tibias. Bones were placed in a 12.3‐mm‐diameter sample holder filled with 75% ethanol and immobilized with Styrofoam. Samples were scanned at 8‐μm resolution (isotropic voxel size) using an energy level of 55 kVp, an integration time of 300 ms, and an intensity of 109 μA. Morphometric parameters were evaluated using a fixed Gaussian filter and a threshold of 220 for trabecular bone and 250 for both cortical bone and whole bone.

**Figure 1:**
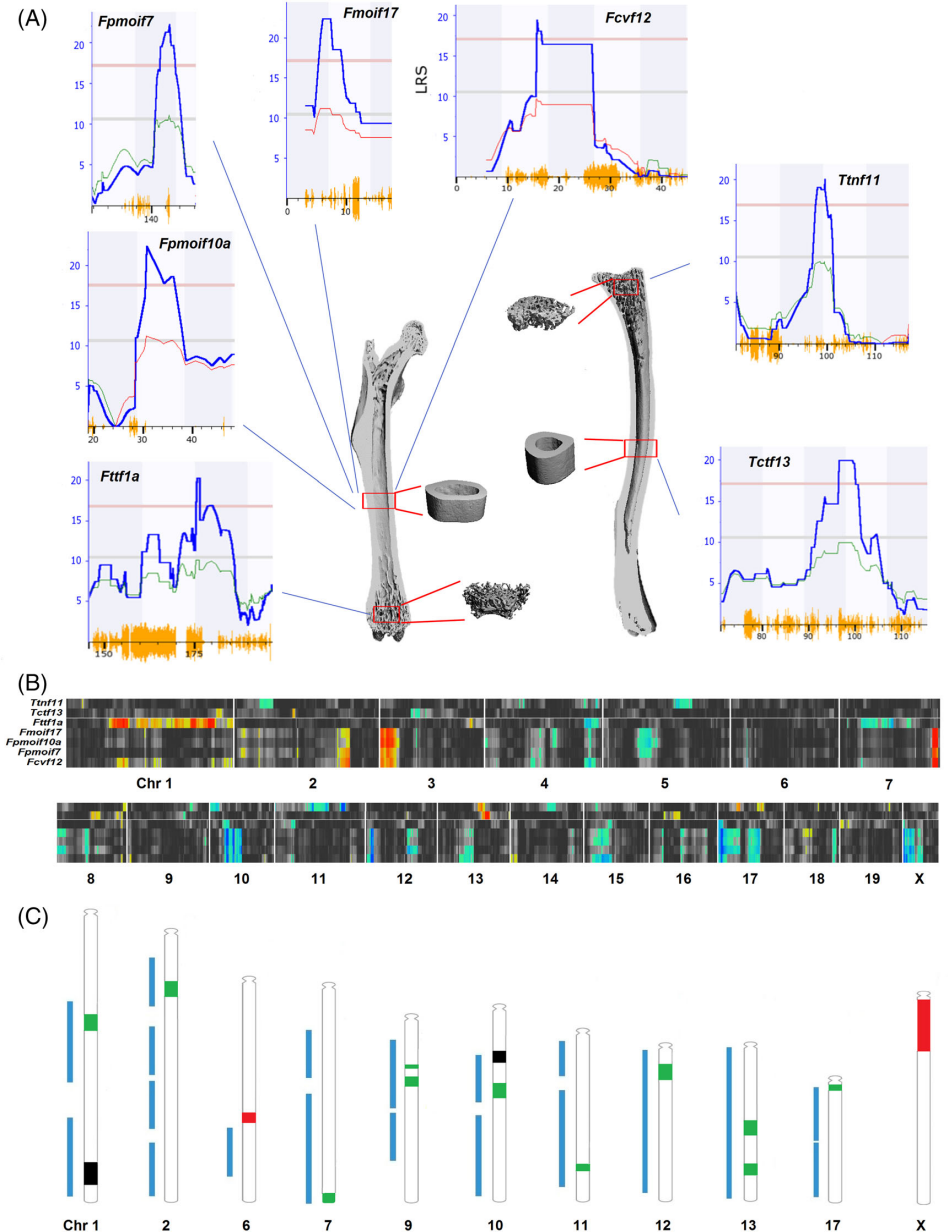
(*A*) Representative μCT image reconstructions of whole bone (cut‐planes of femur on the left and tibia on the right). Four red boxes represent reconstructed microtraits of either cortical bone (midshaft) or trabecular bone (bottom and top, distal femur and proximal tibia) generated from 100 transverse sectional slices. The seven most robust quantitative trait loci (QTL) are shown around the periphery with corresponding trait identifiers and QTL: First letter *F* or *T* (femur or tibia), followed by abbreviation of key bone phenotype, and *f* or *m* (female or male if a QTL was sex‐specific). pmoi = polar moment of inertia; cv = cortical volume; ct = cortical thickness; tn = trabecular number; tt = trabecular thickness. The final number is the chromosome number. For each QTL map, the *x* axis is given in megabases, the left *y* axis is the likelihood ratio statistic (LRS) score. The red horizontal line provides the genome‐wide significance level based on 2000 permutations. Orange hash along the *x* axis indicates SNP density. Heavy blue lines provide linkage statistics, whereas thin green and red lines provide an estimate of the additive genetic effect (right *y* axis).^29^ (*B*) The QTL heat map provides whole‐genome mapping results for all seven phenotypes in the form of color‐coded horizontal bands. Bands of more‐intense color correspond to QTL linkage peaks, and colors encode the additive effect of alleles (blue for *B* and red for *D* alleles). (*C*) Chromosomal ideograms for all 16 significant QTL (chromosomes 1, 2, 6, 7, 9, 10, 11, 12, 13, 17, and X) combined with mouse bone QTL (interval coverage in blue bars) on these chromosomes listed on Rat Genome Database (RGD) from GViewer (*www.rgd.mcw.edu/rgdweb/search/qtls.html?term=bone&chr=ALL&start=&stop=&map=360&rs_term=&vt_term=&speciesType=2&obj=qtl&fmt=5*). *Fttf1b* and *Fpmoif10a* are two QTL with similar phenotypes and map positions (labeled in black). *Ttda6*, *FcvfXa*, and *FcvfXb* are three novel loci that do not overlap those listed in the RGD (labeled in red). All of other 11 QTL overlap some known bone QTL (usually BMD), but are now linked to specific μCT bone traits and are therefore new or refined (labeled in green).

Each femur and tibia were measured separately using Scanco software (Scanco Medical AG, Brüttisellen, Switzerland) that generates more than 50 quantitative traits per bone (whole bone, cortical, and trabecular segments). We selected a subset of 25 of the more interesting and interpretable measurements for in‐depth analysis (Table 1). Three whole‐bone parameters were used: length, mineralized volume, and material bone mineral density (mBMD). For cortical bone, 100 transverse slices were acquired at the middle of the shaft: a total length of 0.8 mm. From these cross‐sections, 11 cortical microtraits were generated, including cortical thickness, cortical volume, porosity, and polar and area moment of inertia. For trabecular bone analysis, 100 slices were acquired at the secondary spongiosa of the distal femur or proximal tibia. Eleven trabecular microtraits were generated, including bone volume fraction (BV/TV), trabecular thickness, trabecular number, trabecular separation, and the trabecular connectivity density (Fig. 1 *A*).

**Table 1:** Abbreviations and Explanations of Bone Traits Measured by μCT

Site	Abbreviation	Explanation
Whole bone	Length (mm)	Length of whole bone (femur or tibia) in the longitudinal axis
	Mineralized.Volume (mm^3^)	Total mineralized volume of femur or tibia
	Material.BMD (mgHA/cm^3^)	Material BMD of whole bone in units of hydroxyapatite density
Cortical bone^a^	Ct.TV (mm^3^)	Total volume of a cortical bone segment of 100 cross‐sectional slices at midshaft
	Ct.BV (mm^3^)	Mineralized volume of a cortical bone segment (based 100 cross‐sectional slices) at midshaft
	Ct.BV/TV (ratio)	Cortical bone fraction (BV/TV)
	Ct.Porosity (%)	Cortical bone porosity = (1 – BV/TV) × 100%
	Ct.Th (mm)	Cortical bone thickness
	Ct.Apparent.BMD (mgHA/ cm^3^)	Cortical apparent bone mineral density in units of hydroxyapatite density
	Ct.Material.BMD (mgHA/ cm^3^)	Cortical material bone mineral density in units of hydroxyapatite density
	CSV (mm^3^)	Total volume of 100 cross‐sectional slices in cortical bone segment, including the marrow space
	Ct.V (mm^3^)	Total mineralized volume of 100 cross‐sectional slices in cortical bone segment
	Ct.Ma.V (mm^3^)	Total marrow volume of 100 cross‐sectional slices = CSV – Ct.V
	CSA (mm^2^)	Cross‐sectional area = CSV/(0.008 × 100)
	Ct.Ar (mm^2^)	Cross‐sectional area of cortical bone = Ct.V/(0.008 × 100)
	Ct.Ma.Ar (mm^2^)	Cross‐sectional area of bone marrow = Ct.Ma.V/(0.008 × 100)
	Ct.pMOI (mm^4^)	Cortical polar moment of inertia
	Ct.Imax (mm^4^)	Cortical moment of inertia around the shorter axis
	Ct.Imin (mm^4^)	Cortical moment of inertia around the longer axis
	Imax/Cmax (mm^3^)	Cortical moment of inertia around the shorter axis divided by the maximum radius perpendicular to Imax direction
	Imin/Cmin (mm^3^)	Cortical moment of inertia around the longer axis divided by the maximum radius perpendicular to Imin direction
Trabecular bone^b^	Trab.TV (mm^3^)	Total volume in trabecular bone segment (based on 100 cross‐sectional contour slices)
	Trab.BV (mm^3^)	Mineralized volume in trabecular bone segment (based on 100 cross‐sectional contour slices)
	Trab.BV/TV (ratio)	Trabecular bone fraction (BV/TV)
	Trab.Conn.Dens. (1/mm^3^)	Connectivity density in trabecular bone, normed by TV
	Trab.SMI	Structure model index in trabecular bone: 0 for parallel plates, 3 for cylindrical rods
	Trab.N (1/mm)	Trabecular number
	Trab.Th (mm)	Trabecular thickness
	Trab.Sp (mm)	Trabecular separation = marrow thickness
	Trab.(1/N).SD	Standard deviation of local inverse number
	Trab.Th.SD	Standard deviation of local thicknesses
	Trab.Sp.SD	Standard deviation of local separations
	Trab.Apparent.BMD (mgHA/cm^3^)	Trabecular apparent bone mineral density in units of hydroxyapatite density
	Trab.Material.BMD (mgHA/cm^3^)	Trabecular material bone mineral density in units of hydroxyapatite density
	Trab.DA (ratio)	Degree of anisotropy in trabecular bone, 1 = isotropic, >1 anisotropic by definition

a All the cortical bone traits are based on 100 transverse slices at the middle shaft of bone cortex.

b All the trabecular bone traits are based on 100 transverse slices at the secondary spongiosa of distal femur or proximal tibia.

### Statistical analysis

Bones were harvested immediately after sacrifice from roughly equal numbers of males and females, ranging from 50 days to 375 days‐of‐age (100 ± 56 SD). This age range is equivalent to adolescent to middle age in humans.^30^ Body weights ranged from 13.4 to 48.5 g (24.9 ± 5.1 SD). The relation between body weight and the logarithm of age fits a linear regression reasonably well in which weight (g) = −3.5 + 14.5 (log_10_ of age), with an *r* = 0.45 and *p* < 0.0001.

All data and metadata on cases used in this study are provided in the Supplementary Data S1. To minimize effects of age as a confounder, we performed linear regression for each variable across 307 male and 290 female samples separately, using logarithm of age as a predictor. Log age‐corrected values were computed by adding the residuals to means for male or female samples separately (Supplementary Data S1, Sheet Female_Raw_Res_Corr and Male_Raw_Res_Corr). Because the average age of males and females was approximately 100 days, the corrected values by case, strain, and sex should be considered as those that will typically be measured at this age. Both the original values and the corrected values are provided in Supplementary Data S1. Sex‐averaged values were computed as above by fitting the cofactors sex and logarithm of age (without grouping by strain) across the entire data set. Means were added to the residuals, and these values were summarized to generate sex‐ and age‐corrected strain means.

We also analyzed the relation between body weight and bone length/volume before and after correction for age. As expected, there is a strong positive correlation before age correction; body weight and bone volume covary with an *r* of 0.61 when both sexes are combined. After the log‐age correction, there is still a significant association between body weight and bone parameters. For example, the correlation between femur volume and body weight is 0.35. We chose not to correct for this source of variation in subsequent analyses because body weight and size are also key variables of interest. But this does mean that bone data need to be considered in light of general variation in body size. To determine the body‐size effects on mapping after eliminating age as a cofactor, we mapped using both data adjusted by logarithm of age only and data adjusted by logarithm of age plus body weight for all traits associated with robust QTL. Effects of sex and strain as predictors were estimated by ANOVA.

After generating cleaned and adjusted values, we again searched for outliers at both the level of individual cases and strain means. A few outlier cases and strains (e.g., BXD13 and BXD78) were censored in some analyses as described in the Results section and the figure legends, either by complete removal or by winsorizing outliers.^31^ Mean strain data entered into GeneNetwork (GN) have been reviewed, and when necessary, have also been winsorized. However, we do provide the original value in trait descriptions. Users can revert to the original data as needed.

Heritability (*h* ^2^) was calculated for all key traits. The variance and bias of the estimate of *h* ^2^ was computed using a jackknife in JMP Pro 12 (SAS Institute, Inc., Cary, NC, USA).^32^, ^33^ This involved calculating heritabilities for subsamples of data, each missing all data for one strain (*h*_(−*i)*_). The jackknife variance is

$$V_{\mathrm{JK}}\left(h^{2}\right)=\frac{n-1}{n}\sum_{i=1}^{n}{\left(h_{\left(-i\right)}-{\bar{h}}_{\left(\cdot \right)}\right)}^{2}$$

where ${\bar{h}}_{\left(\cdot \right)}$ is the mean heritability using all strains.

To test for sex‐difference heritabilities, we computed the *Z* value:

$$z=\frac{{\bar{x}}_{1}-{\bar{x}}_{2}}{\sqrt{\frac{\sigma_{1}^{2}}{n_{1}}+\frac{\sigma_{2}^{2}}{n_{2}}}}$$

where ${\bar{x}}_{1}$ and ${\bar{x}}_{2}$ are the averages of the female and male heritabilities of each bone trait; *σ*_1_^2^ and *σ*_2_^2^ are the jackknife variances of female and male heritabilities, *n*_1_ = *n*_2_ = 35. |*z*| ≥ 3.18 is considered significant with a Bonferroni‐corrected *p* of <0.00143 (0.05/35 ≅ 0.00143, two‐tailed).

### Correlation analysis

We studied correlations among bone phenotypes using strain averages. We selected three representative traits for each of three major categories: (1) whole bone (GN Record IDs 18130, 18131, 18132), (2) cortical bone (GN 18134, 18136, 18141), and (3) trabecular bone (GN 18146, 18148, 18149). Because the sample size is reasonably large (*n* = 63), we used Pearson product–moment correlations, and confirmed results were not sensitive to outliers. Finally, we computed correlations between bone microtraits and a large femur mRNA expression data set (UCLA GSE27483 *BXD Bone Femur ILM Mouse WG‐6 v1.1 (Jan13) RSN* in GN (GN accession number: GN410). This expression data set was generated by Farber and colleagues^17^, ^34^ and includes data for 32 BXD strains and many other strains for which we have matched phenotypes. Because the overlap of sample size is modest, we used rank‐order correlations to compare bone phenotypes with expression data.

### QTL mapping

We initially carried out conventional interval mapping using Haley‐Knott regression equations^35^ as implemented in GN. To estimate genome‐wide thresholds of significance, we permuted phenotypes 2000 times. CIs were defined as the chromosomal region within a 1.5 LOD drop from the linkage peak. We have taken several approaches to evaluate the consistency of QTL results, including the use of two genotype files, different methods to correct for age and sex, and different mapping algorithms that handle kinship relations (see below). In total, we mapped 50 traits for males, females, and for sex‐averaged means. Initial analysis was corrected for age, but for comparison, we also remapped using young animals within a relatively narrow age range. All aspects of this analysis can be reviewed, replicated, and extended using the GN record IDs in Table 4 and Supplementary Data S1.

#### *Classic and new genotype files*

We used two genotype files for mapping. The first is a file that has been used by almost all investigators from 2002 through to late 2016. We refer to this as the “classic” file because it has been used in hundreds of studies. The second file was released in January 2017 and includes roughly twice as many markers. Both files are available at www.genenetwork.org/webqtl/main.py?FormID=sharinginfo&GN_AccessionId=600.

The two files are similar. The main difference is that several strains were not yet fully inbred during the earlier phase of genotyping, but are now fully inbred. Because the cases we have studied here were born between 2011 and 2013, it is useful to compare results using both files.

#### *Age effects*

We were concerned that cases older than 150 days may have had sufficiently different bone architecture that the statistical age adjustment would not fully compensate. For this reason, we compared results based on the complete data set for all 597 cases corrected for log age (GN 18130 to 18279) to a subset of 484 cases ranging from 65 to 116 days and processed without any age correction (GN 18986 to 19086). This age range is equivalent to young adults in humans.^30^

#### *Mapping algorithms*

Differences in algorithms and their sensitivities to trait distribution and kinship among strains will have effects on mapping results. We therefore remapped traits, in particular the 13 traits that gave strong initial results, using complementary algorithms. These methods include variants of R/qtl,^36^, ^37^, ^38^ and two algorithms that explicitly model kinship, pyLMM^39^ and GEMMA.^40^, ^41^ All algorithms were run using implementations that are part of GeneNetwork 2 (gn2.genenetwork.org*)*.

#### *Composite interval mapping*

For traits with multiple QTL, we wanted to ensure that the loci were not in statistical linkage.^42^ To ensure independence, we selected background control markers close to one of the QTL peaks and remapped using composite interval mapping methods.

### Gene ontology scoring system of candidate genes

The 16 QTL we have mapped each contain between 45 to 174 positional candidate genes within the 1.5 LOD CIs, and collectively include 1638 protein‐coding genes. Based on our survey of the literature, only 2% (36) have been previously linked to bone biology. To evaluate the remaining 98% of candidates for possible association with bone biology, we developed an efficient, comprehensive, and quantitative method that generates an objective bone score using methods similar to those described in previous work by us and others.^26^, ^27^, ^43^ We specifically defined a bone score that estimates the potential association of each gene to bone biology when compared against a reference set of 770 genes already well‐known to be associated with 34 bone gene ontology (GO) terms. We exploited the same femur‐expression data set (GN410) that includes probes that target essentially all protein‐coding transcripts. For each of 46,621 probes, we first calculated the absolute Spearman correlation between the mRNA abundance of the gene and all other transcripts. We then selected the top 1000 probes with the highest correlations and performed a GO enrichment analysis (biological process) based on the hypergeometric test^44^, ^45^, ^46^:

$$p=1-\sum_{i=0}^{k-1}\frac{\left(\begin{array}{c} m\\ i \end{array}\right)\left(\begin{array}{c} M-m\\ N-i \end{array}\right)}{\left(\begin{array}{c} M\\ N \end{array}\right)}$$

where *M* represents the total number of genes targeted by all probe IDs (*n* = 30,880); *N* represents the number of unique genes among the top 1000 covariates; *m* represents the number of genes listed in the GO term; and *k* represents the number of genes among the top 1000 covariates that are in the GO term.

For example, the top 1000 covariates of *Alpl* (alkaline phosphatase, probe ID ILM2340168) include 369 genes associated with “ossification” (GO:0001503). Even after correction for multiple comparisons, the *p* value of this GO‐term enrichment is 5 × 10^−23^. We computed enrichment *p* values for a set of 34 bone‐associated GO terms (Supplementary Data S2). The average *p* value of 40 well‐known bone genes such as *Alpl* was used as a reference standard against which we compared all other genes/probes (also see Supplementary Data S2). Many candidate genes had *p* values that were as good as or better than those of these 369 known bone‐associated genes. We converted values to –log_10_ (*p*) across all 34 terms and used the average value as a GO‐associated bone score. Genes such as *Alpl* that are linked to several bone GO terms typically have scores above 1 (the peak score is 10.5 for *Col15a1*). Genes linked to 10 or more GO terms typically have scores above 2. Genes without known links to bone have averages well below 1. A large subset of genes was further defined as members of the bone ignorome: genes that have unusually high bone scores based on this analysis, but that have no known literature associated with the skeletal system.

Finally, we generated a summary candidate score on a scale of 1 to 10 points for all genes based on:

1. Average bone score (1 to 3). The gene is assigned 1 point if its average bone score across GOs is between 0 and 1; 2 points if between 1 and 3; and 3 points if >3.
2. Highest bone score (0 to 1). Some genes have high GO scores for one or two of the 34 GOs. We therefore assigned these genes 1 point, provided that its highest bone score was between 5 and 10.
3. Coding DNA variants (0 to 3). In a recent study we defined 35,068 coding SNPs in the BXD family, of which 11,979 are nonsynonymous (nsSNP) with high Grantham scores (complete list is given in Supplementary Data S4 of Wang et al.^20^ A gene is assigned 1 point if the sum of Grantham values is <100, 2 if the sum is 100 to 300, and 3 if >300. We also identified 173 SNPs associated with nonsense, frame shift, or splice site mutation (Supplementary Data S5 of Wang et al.^20^ Genes with any of these types of variants were assigned 3 points.
4. Cis‐regulation (0 to 3). Genes with strong evidence of cis‐acting control (a so‐called cis eQTL) in bone (*BXD Bone Femur ILM Mouse WG‐6 v1, v1.1* (Jan 13) RSN, GeneNetwork Accession Number: GN410)^17^, ^34^ were assigned 3 points. Genes with cis eQTL in cartilage (UCLA BXD Cartilage, GN Accession Number: GN178) or muscle (EPFL/LISP BXD CD Muscle Affy Mouse Gene 1.0 ST (Nov12), GN Accession Number: GN397) were assigned 2 points. Genes with cis eQTL in any other tissue were assigned 1 point.

All candidate genes received a summary candidate score on a 0 to 10 point scale (Supplementary Data S3 and Table 5). We focused analysis on a subset of 212 candidates within 16 QTL with scores greater than 4. With the long‐term goal of functional validating some of these candidates, we shortened this list further, and therefore restricted more detailed analysis to the strongest 50 selected across seven robust QTL.

### Candidate gene analysis

In addition to NCBI PubMed, we evaluated candidates using data extracted from four major resources:

1. The Rat Genome Database (RGD, www.rgd.mcw.edu). As of our search, RGD provided a list of 855 genes linked to bone biology across human, rat, and mouse.

2. The Human GWAS Gene Compendium (www.ebi.ac.uk/gwas), using “bone” and “skeletal” as key words at a *p* threshold ≤5 × 10^−4^. We downloaded a list of 1125 genes associated with diseases that impact bone and the skeletal system.

3. The International Mouse Phenotyping Consortium (www.mousephenotype.org), a collection of phenotype data on mouse knockout lines. We obtained a set of 699 genes associated with abnormal skeletal phenotype (mouse phenotype [MP]: 0005508).

4. Using the 2017 UK BioBank (UKBB) eBMD GWAS Summary Statistics data,^47^ we defined bins between the upstream and downstream SNPs with a linkage disequilibrium *r* ^*2*^ of ≥0.7 with the lead SNP, and calculated from European populations in the 1000 Genomes Phase III data.^48^ For each bin, we identified all overlapping genes. If no genes intersected a bin, the nearest upstream and downstream genes were included. This yielded 731 genes underlying GWAS loci for eBMD (Supplementary Data S3, Sheet 731_BMD_GWAS_genes).

## Results

**Figure 2:**
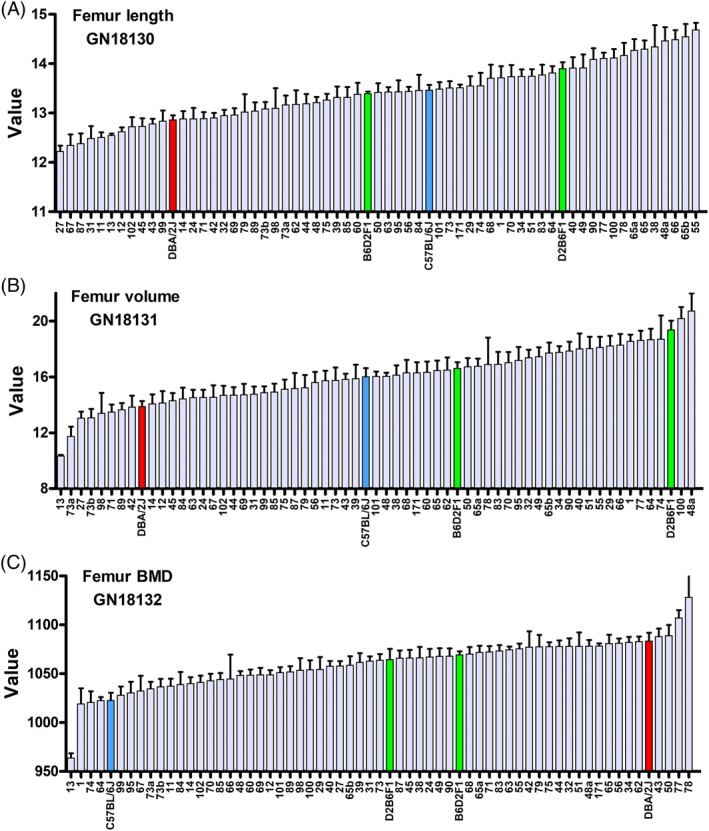
Representative femur traits (sex‐averaged) of two parental strains (B6 and D2), reciprocal F1 hybrids (B6D2F1 and D2B6F1), and 59 BXD strains (mean ± SEM). (*A*) Femur length (GN 18130). (*B*) Femur volume (GN 18131). (*C*) Femur BMD (GN 18132). Note that BXD13 is an outlier and requires special handling. We opted to winsorize this value in GN from 963 to 1016 mgHA/cm^3^.

Variation among strains was moderate to high for almost all bone traits. This included traits such as length, mineralized volume, and material BMD, as well as cortical and trabecular traits, such as thickness, porosity, and polar moment at inertia (Table 2; GN IDs 18130 to 18279, and Supplementary Data S1). For example, femoral length ranged from 12.2 ± 0.1 mm in BXD27 to 14.7 ± 0.13 mm in BXD55. In contrast, the range of variation of BMD was much less (only 1.7% range). Variation in strain averages was of course much more modest than variation among the entire pool of individuals (Fig. 2). Typical coefficients of variation (CVs) for traits at the individual level ranged from a high of 34% for femur trabecular connectivity density to a low of 2% for tibia material BMD. For example, the threefold difference in bone volume between extreme cases was reduced to 1.5‐fold (13.1 versus 20.2 mm) when considering strain means.

**Table 2:** Summary of Femur Phenotypes With Heritability

Sex	Strain	*N*	Femur length (mm)	Femur volume (mm^3^)	Femur BMD (mgHA/cm^3^)	Femur cortical porosity (%)	Femur cortical thickness (mm)	Femur cortical pMOI (mm^4^)	Femur trabecular bone fraction (BV/TV, %)	Femur trabecular connectivity density (1/mm^3^)	Femur trabecular thickness (mm)
F	C57BL/6J	6	13.529 ± 0.176	14.178 ± 0.374	997.391 ± 5.974	1.964 ± 0.164	0.174 ± 0.008	0.224 ± 0.053	0.069 ± 0.014	97.514 ± 27.759	0.037 ± 0.001
M	C57BL/6J	12	13.481 ± 0.087	17.453 ± 0.903	1035.563 ± 8.803	1.672 ± 0.105	0.200 ± 0.009	0.319 ± 0.036	0.144 ± 0.026	179.605 ± 26.851	0.043 ± 0.002
F	DBA/2 J	13	12.822 ± 0.080	13.604 ± 0.538	1082.959 ± 14.005	1.578 ± 0.101	0.216 ± 0.006	0.165 ± 0.031	0.087 ± 0.014	109.611 ± 15.173	0.040 ± 0.002
M	DBA/2 J	13	12.880 ± 0.086	14.073 ± 0.322	1085.283 ± 8.003	1.498 ± 0.047	0.214 ± 0.007	0.161 ± 0.012	0.124 ± 0.011	227.495 ± 25.913	0.042 ± 0.001
F	D2B6F1	3	13.752 ± 0.036	17.306 ± 1.147	1060.429 ± 26.612	1.653 ± 0.341	0.225 ± 0.001	0.355 ± 0.072	0.083 ± 0.027	89.568 ± 33.391	0.042 ± 0.002
M	D2B6F1	4	14.017 ± 0.212	21.136 ± 0.429	1068.421 ± 5.405	1.269 ± 0.039	0.255 ± 0.007	0.493 ± 0.028	0.191 ± 0.035	188.315 ± 23.723	0.049 ± 0.004
F	B6D2F1	3	13.441 ± 0.059	15.007 ± 0.352	1069.966 ± 2.365	1.369 ± 0.068	0.201 ± 0.005	0.240 ± 0.007	0.138 ± 0.014	187.241 ± 28.057	0.044 ± 0.001
M	B6D2F1	4	13.331 ± 0.071	18.404 ± 0.873	1075.654 ± 7.314	1.313 ± 0.121	0.246 ± 0.010	0.364 ± 0.030	0.250 ± 0.025	279.165 ± 58.211	0.052 ± 0.004
F	BXD1	4	13.811 ± 0.452	17.079 ± 0.770	1021.392 ± 33.490	1.599 ± 0.145	0.211 ± 0.023	0.346 ± 0.025	0.073 ± 0.028	117.936 ± 49.043	0.041 ± 0.001
M	BXD1	5	13.677 ± 0.243	19.924 ± 0.564	1017.718 ± 13.515	1.313 ± 0.103	0.224 ± 0.011	0.441 ± 0.016	0.157 ± 0.025	158.062 ± 48.683	0.061 ± 0.008
F	BXD11	3	12.376 ± 0.175	13.672 ± 0.723	1025.773 ± 9.838	1.283 ± 0.081	0.202 ± 0.003	0.252 ± 0.017	0.095 ± 0.009	87.859 ± 7.838	0.045 ± 0.002
M	BXD11	3	12.601 ± 0.089	17.700 ± 0.849	1050.523 ± 2.341	0.915 ± 0.077	0.251 ± 0.011	0.320 ± 0.025	0.237 ± 0.021	241.900 ± 30.241	0.052 ± 0.004
F	BXD12	4	12.591 ± 0.065	11.952 ± 0.353	1055.507 ± 6.712	1.502 ± 0.046	0.189 ± 0.004	0.251 ± 0.017	0.035 ± 0.007	18.369 ± 7.544	0.031 ± 0.001
M	BXD12	4	12.635 ± 0.158	16.234 ± 1.476	1043.468 ± 4.183	1.614 ± 0.304	0.221 ± 0.012	0.375 ± 0.058	0.186 ± 0.012	131.679 ± 10.439	0.049 ± 0.001
F	BXD13	0	N/A	N/A	N/A	N/A	N/A	N/A	N/A	N/A	N/A
M	BXD13	2	12.649 ± 0.029	11.425 ± 0.050	963.190 ± 4.230	1.736 ± 0.030	0.167 ± 0.001	0.185 ± 0.005	0.068 ± 0.001	121.523 ± 8.509	0.033 ± 0.001
F	BXD14	3	12.489 ± 0.053	12.528 ± 0.181	1032.288 ± 5.357	1.599 ± 0.084	0.186 ± 0.005	0.225 ± 0.004	0.089 ± 0.003	141.212 ± 13.973	0.036 ± 0.001
M	BXD14	3	13.098 ± 0.150	14.927 ± 1.421	1041.235 ± 10.842	1.700 ± 0.113	0.194 ± 0.006	0.320 ± 0.047	0.121 ± 0.010	174.946 ± 25.227	0.039 ± 0.001
F	BXD24	2	12.224 ± 0.021	12.727 ± 0.127	1073.875 ± 13.213	1.209 ± 0.104	0.230 ± 0.001	0.177 ± 0.010	0.106 ± 0.003	124.989 ± 4.329	0.041 ± 0.000
M	BXD24	2	13.006 ± 0.079	14.751 ± 0.480	1067.121 ± 25.922	1.071 ± 0.140	0.261 ± 0.004	0.372 ± 0.142	0.132 ± 0.017	193.775 ± 31.600	0.042 ± 0.002
F	BXD27	3	12.266 ± 0.134	13.662 ± 0.461	1063.378 ± 4.214	1.484 ± 0.121	0.192 ± 0.006	0.277 ± 0.021	0.128 ± 0.026	192.388 ± 51.902	0.043 ± 0.003
M	BXD27	3	12.250 ± 0.079	12.671 ± 0.366	1050.160 ± 2.985	1.739 ± 0.315	0.186 ± 0.004	0.238 ± 0.008	0.099 ± 0.003	150.561 ± 11.935	0.041 ± 0.002
F	BXD29	2	13.192 ± 0.359	17.558 ± 0.607	1064.533 ± 18.882	1.924 ± 0.393	0.223 ± 0.002	0.410 ± 0.012	0.207 ± 0.022	235.793 ± 30.098	0.051 ± 0.002
M	BXD29	6	13.966 ± 0.210	20.357 ± 1.284	1044.187 ± 21.407	1.793 ± 0.121	0.204 ± 0.007	0.416 ± 0.047	0.230 ± 0.019	306.409 ± 26.746	0.047 ± 0.002
F	BXD31	1	N/A	N/A	N/A	N/A	N/A	N/A	0.052	52.690	0.034
M	BXD31	4	12.253 ± 0.058	15.371 ± 0.500	1064.321 ± 5.739	1.232 ± 0.072	0.235 ± 0.012	0.355 ± 0.038	0.193 ± 0.024	160.110 ± 18.904	0.057 ± 0.003
F	BXD32	6	12.927 ± 0.209	15.783 ± 0.573	1060.550 ± 9.021	1.335 ± 0.054	0.224 ± 0.002	0.220 ± 0.003	0.173 ± 0.013	191.970 ± 12.952	0.049 ± 0.002
M	BXD32	7	13.116 ± 0.122	19.051 ± 0.880	1091.380 ± 8.543	1.120 ± 0.033	0.258 ± 0.005	0.368 ± 0.031	0.238 ± 0.023	301.690 ± 20.993	0.051 ± 0.002
F	BXD34	12	13.658 ± 0.197	16.317 ± 0.513	1084.060 ± 8.190	1.640 ± 0.064	0.192 ± 0.003	0.187 ± 0.004	0.107 ± 0.004	151.223 ± 8.159	0.042 ± 0.001
M	BXD34	10	13.871 ± 0.183	19.293 ± 0.676	1080.580 ± 5.769	1.409 ± 0.046	0.213 ± 0.002	0.331 ± 0.014	0.191 ± 0.013	272.218 ± 21.465	0.045 ± 0.001
F	BXD38	0	N/A	N/A	N/A	N/A	N/A	N/A	N/A	N/A	N/A
M	BXD38	4	14.390 ± 0.430	17.132 ± 0.662	1066.637 ± 10.651	1.362 ± 0.055	0.207 ± 0.005	0.271 ± 0.021	0.169 ± 0.016	267.982 ± 36.139	0.040 ± 0.002
F	BXD39	2	14.299 ± 0.313	15.526 ± 0.084	1013.768 ± 4.030	1.715 ± 0.270	0.188 ± 0.016	0.302 ± 0.059	0.219 ± 0.115	251.840 ± 113.506	0.052 ± 0.007
M	BXD39	9	13.191 ± 0.216	16.795 ± 1.275	1071.651 ± 8.359	1.525 ± 0.109	0.198 ± 0.006	0.315 ± 0.027	0.204 ± 0.027	283.661 ± 27.968	0.046 ± 0.003
F	BXD40	3	14.070 ± 0.612	17.800 ± 2.578	1056.463 ± 16.050	1.389 ± 0.067	0.194 ± 0.004	0.305 ± 0.017	0.081 ± 0.018	96.700 ± 29.625	0.042 ± 0.002
M	BXD40	7	13.964 ± 0.273	19.140 ± 1.561	1058.628 ± 6.208	1.639 ± 0.099	0.197 ± 0.010	0.319 ± 0.032	0.202 ± 0.021	290.189 ± 24.561	0.048 ± 0.003
F	BXD42	3	12.888 ± 0.119	13.078 ± 1.023	1093.227 ± 5.936	1.536 ± 0.098	0.192 ± 0.010	0.268 ± 0.016	0.067 ± 0.016	101.117 ± 33.642	0.043 ± 0.003
M	BXD42	1	12.868	14.041	1031.472	1.499	0.203	0.322	0.156	213.694	0.047
F	BXD43	5	12.523 ± 0.073	14.277 ± 0.312	1091.343 ± 13.418	1.771 ± 0.108	0.193 ± 0.003	0.210 ± 0.009	0.141 ± 0.007	192.914 ± 14.680	0.042 ± 0.001
M	BXD43	5	12.898 ± 0.122	17.262 ± 0.770	1091.088 ± 11.084	1.498 ± 0.073	0.220 ± 0.004	0.320 ± 0.029	0.161 ± 0.038	203.937 ± 56.161	0.045 ± 0.002
F	BXD44	5	13.033 ± 0.289	13.269 ± 0.734	1080.821 ± 9.563	1.539 ± 0.080	0.197 ± 0.009	0.220 ± 0.013	0.124 ± 0.017	151.634 ± 14.036	0.043 ± 0.003
M	BXD44	4	13.391 ± 0.217	16.179 ± 0.858	1075.058 ± 5.399	1.191 ± 0.063	0.233 ± 0.005	0.281 ± 0.016	0.182 ± 0.031	250.397 ± 29.664	0.045 ± 0.003
F	BXD45	7	12.488 ± 0.132	12.916 ± 0.586	1071.149 ± 11.202	1.897 ± 0.138	0.184 ± 0.006	0.245 ± 0.063	0.136 ± 0.010	218.080 ± 22.486	0.040 ± 0.001
M	BXD45	1	12.354	13.463	1053.226	1.426	0.184	0.242	0.078	114.577	0.036
F	BXD48	3	13.101 ± 0.180	14.833 ± 0.481	1048.655 ± 6.990	1.521 ± 0.119	0.189 ± 0.009	0.282 ± 0.018	0.108 ± 0.015	135.128 ± 16.121	0.042 ± 0.002
M	BXD48	4	13.198 ± 0.248	17.145 ± 0.380	1042.273 ± 5.258	1.296 ± 0.037	0.219 ± 0.007	0.409 ± 0.024	0.176 ± 0.034	163.769 ± 12.461	0.048 ± 0.005
F	BXD48a	3	14.796 ± 0.092	21.273 ± 0.552	1084.702 ± 6.935	1.709 ± 0.020	0.202 ± 0.007	0.318 ± 0.024	0.162 ± 0.013	212.449 ± 35.886	0.046 ± 0.002
M	BXD48a	1	14.547	21.756	1075.341	2.259 ± 0.550	0.168 ± 0.042	0.265 ± 0.029	0.171	218.479	0.047
F	BXD49	5	13.889 ± 0.442	16.029 ± 0.672	1064.274 ± 9.073	1.437 ± 0.106	0.203 ± 0.007	0.377 ± 0.027	0.098 ± 0.011	151.320 ± 18.885	0.039 ± 0.002
M	BXD49	3	14.113 ± 0.171	19.560 ± 1.444	1076.908 ± 19.370	1.875 ± 0.378	0.229 ± 0.005	0.643 ± 0.060	0.177 ± 0.025	248.064 ± 29.315	0.047 ± 0.002
F	BXD50	4	13.273 ± 0.177	15.707 ± 0.834	1089.084 ± 10.494	1.517 ± 0.070	0.201 ± 0.007	0.260 ± 0.005	0.071 ± 0.006	76.985 ± 21.575	0.041 ± 0.001
M	BXD50	2	13.722 ± 0.446	17.743 ± 0.835	1089.678 ± 31.016	1.205 ± 0.093	0.218 ± 0.014	0.326 ± 0.001	0.153 ± 0.007	257.088 ± 39.999	0.043 ± 0.000
F	BXD51	3	13.675 ± 0.176	17.399 ± 1.047	1070.744 ± 23.819	1.214 ± .0140	0.223 ± 0.004	0.249 ± 0.028	0.164 ± 0.019	292.273 ± 33.112	0.042 ± 0.002
M	BXD51	2	13.847 ± 0.285	18.443 ± 1.639	1090.018 ± 0.966	1.213 ± 0.145	0.224 ± 0.011	0.274 ± 0.038	0.174 ± 0.009	344.364 ± 20.297	0.041
F	BXD55	1	14.972	13.411	1094.549	2.138	0.158	0.195	0.061	98.457	0.031
M	BXD55	5	14.702 ± 0.131	19.893 ± 0.327	1071.538 ± 4.306	1.477 ± 0.054	0.199 ± 0.003	0.267 ± 0.009	0.170 ± 0.006	229.121 ± 12.619	0.048 ± 0.001
F	BXD56	3	13.643 ± 0.100	16.666 ± 0.334	1083.707 ± 5.038	1.361 ± 0.127	0.203 ± 0.002	0.261 ± 0.007	0.132 ± 0.010	209.582 ± 28.704	0.040 ± 0.000
M	BXD56	4	13.274 ± 0.069	14.866 ± 0.572	1085.606 ± 6.553	1.488 ± 0.064	0.216 ± 0.006	0.276 ± 0.021	0.132 ± 0.015	268.948 ± 10.629	0.039 ± 0.002
F	BXD60	5	12.976 ± 0.463	13.391 ± 1.188	1038.254 ± 10.787	1.542 ± 0.054	0.180 ± 0.010	0.216 ± 0.027	0.120 ± 0.007	170.330 ± 21.245	0.042 ± 0.001
M	BXD60	10	13.549 ± 0.220	18.203 ± 0.835	1055.096 ± 5.896	1.471 ± 0.101	0.208 ± 0.004	0.326 ± 0.020	0.185 ± 0.010	301.356 ± 9.131	0.044 ± 0.001
F	BXD62	5	12.735 ± 0.402	14.524 ± 0.951	1080.138 ± 7.689	1.287 ± 0.108	0.215 ± 0.006	0.316 ± 0.015	0.095 ± 0.010	111.789 ± 15.417	0.045 ± 0.001
M	BXD62	4	13.728 ± 0.182	18.652 ± 1.525	1087.383 ± 5.525	1.179 ± 0.029	0.256 ± 0.007	0.469 ± 0.054	0.154 ± 0.059	178.385 ± 61.682	0.052 ± 0.007
F	BXD63	6	13.367 ± 0.092	13.952 ± 0.547	1075.510 ± 3.753	1.791 ± 0.086	0.194 ± 0.004	0.192 ± 0.010	0.134 ± 0.005	190.611 ± 8.014	0.043 ± 0.001
M	BXD63	2	13.588 ± 0.245	14.178 ± 1.088	1072.410 ± 2.549	1.343 ± 0.105	0.228 ± 0.008	0.253 ± 0.029	0.163 ± 0.010	347.890 ± 2.173	0.039 ± 0.002
F	BXD64	1	13.741	18.525	1024.623	1.176	0.206	0.282	0.259	336.811	0.050
M	BXD64	1	13.893	18.760	1021.347	1.708	0.193	0.302	0.183	398.234	0.043
F	BXD65	9	14.598 ± 0.244	17.185 ± 0.927	1096.500 ± 9.908	1.355 ± 0.096	0.215 ± 0.007	0.256 ± 0.026	0.140 ± 0.017	193.212 ± 29.639	0.046 ± 0.002
M	BXD65	10	14.034 ± 0.163	15.868 ± 0.674	1068.015 ± 12.229	1.453 ± 0.111	0.213 ± 0.008	0.268 ± 0.030	0.153 ± 0.019	234.292 ± 23.582	0.045 ± 0.002
F	BXD65a	4	14.438 ± 0.222	16.252 ± 0.803	1077.536 ± 10.469	1.521 ± 0.226	0.196 ± 0.007	0.272 ± 0.011	0.107 ± 0.012	177.243 ± 9.820	0.041 ± 0.001
M	BXD65a	2	14.393 ± 0.462	17.122 ± 0.562	1065.767 ± 3.935	1.411 ± 0.040	0.207 ± 0.005	0.303 ± 0.002	0.145 ± 0.007	260.315 ± 11.056	0.043 ± 0.002
F	BXD65b	10	14.515 ± 0.314	17.182 ± 0.659	1045.131 ± 8.809	1.666 ± 0.138	0.198 ± 0.005	0.207 ± 0.025	0.119 ± 0.013	210.753 ± 28.777	0.040 ± 0.001
M	BXD65b	5	14.554 ± 0.398	17.694 ± 1.625	1088.247 ± 11.776	1.375 ± 0.082	0.218 ± 0.004	0.244 ± 0.011	0.123 ± 0.020	210.841 ± 43.201	0.040 ± 0.002
F	BXD66	1	14.185	16.236	1005.265	1.482	0.177	0.231	0.092	171.503	0.040
M	BXD66	2	14.695 ± 0.087	19.813 ± 1.055	1064.586 ± 24.540	1.179 ± 0.063	0.225 ± 0.009	0.296 ± 0.065	0.211 ± 0.023	270.819 ± 88.948	0.055 ± 0.012
F	BXD67	4	12.134 ± 0.132	14.119 ± 0.193	1062.413 ± 10.738	0.916 ± 0.067	0.224 ± 0.003	0.320 ± 0.020	0.110 ± 0.002	154.176 ± 1.802	0.046 ± 0.001
M	BXD67	2	12.271 ± 0.158	13.665 ± 0.089	981.907 ± 0.488	1.422 ± 0.003	0.213 ± 0.002	0.359 ± 0.030	0.172 ± 0.017	276.614 ± 15.814	0.043 ± 0.002
F	BXD68	8	14.062 ± 0.162	16.638 ± 0.833	1081.137 ± 4.319	1.330 ± 0.057	0.228 ± 0.004	0.213 ± 0.009	0.095 ± 0.009	92.893 ± 11.648	0.049 ± 0.002
M	BXD68	4	12.912 ± 0.540	14.452 ± 1.270	1050.424 ± 14.848	1.377 ± 0.095	0.220 ± 0.015	0.271 ± 0.049	0.128 ± 0.024	206.433 ± 34.482	0.043 ± 0.004
F	BXD69	6	13.146 ± 0.248	14.449 ± 1.563	1036.981 ± 16.560	1.763 ± 0.171	0.185 ± 0.008	0.201 ± 0.017	0.159 ± 0.031	229.544 ± 38.046	0.044 ± 0.003
M	BXD69	7	12.837 ± 0.102	14.892 ± 0.956	1053.375 ± 4.766	1.300 ± 0.059	0.225 ± 0.004	0.320 ± 0.027	0.242 ± 0.014	336.703 ± 22.542	0.047 ± 0.001
F	BXD70	6	13.310 ± 0.394	14.403 ± 1.094	1053.919 ± 9.451	1.499 ± 0.098	0.195 ± 0.007	0.250 ± 0.032	0.076 ± 0.014	112.349 ± 24.547	0.038 ± 0.002
M	BXD70	6	14.188 ± 0.062	19.633 ± 0.310	1032.436 ± 7.707	1.514 ± 0.065	0.210 ± 0.006	0.330 ± 0.016	0.120 ± 0.007	190.333 ± 16.610	0.040 ± 0.002
F	BXD71	8	12.858 ± 0.171	12.850 ± 0.442	1075.356 ± 7.222	1.591 ± 0.070	0.188 ± 0.005	0.186 ± 0.008	0.049 ± 0.005	37.426 ± 10.861	0.039 ± 0.001
M	BXD71	3	12.893 ± 0.127	13.386 ± 1.516	1066.489 ± 5.858	1.487 ± 0.108	0.223 ± 0.014	0.252 ± 0.033	N/A	N/A	N/A
F	BXD73	3	13.116 ± 0.329	11.000 ± 0.084	1054.770 ± 12.976	1.671 ± 0.152	0.188 ± 0.010	0.184 ± 0.008	0.071 ± 0.002	100.178 ± 17.477	0.037 ± 0.003
M	BXD73	7	13.766 ± 0.143	18.303 ± 1.093	1068.207 ± 8.100	1.526 ± 0.086	0.222 ± 0.005	0.338 ± 0.020	0.213 ± 0.022	250.224 ± 20.592	0.049 ± 0.002
F	BXD73a	2	13.531 ± 0.400	10.893 ± 0.230	1027.813 ± 18.717	2.136 ± 0.292	0.155 ± 0.012	0.154 ± 0.007	0.061 ± 0.017	125.582 ± 50.067	0.032 ± 0.002
M	BXD73a	8	13.089 ± 0.223	12.148 ± 0.737	1033.195 ± 7.776	1.696 ± 0.086	0.189 ± 0.005	0.207 ± 0.012	0.087 ± 0.009	134.396 ± 18.379	0.038 ± 0.002
F	BXD73b	6	12.669 ± 0.179	10.703 ± 0.688	1024.722 ± 12.447	1.679 ± 0.110	0.160 ± 0.005	0.123 ± 0.005	0.104 ± 0.015	210.268 ± 57.296	0.036 ± 0.001
M	BXD73b	5	13.302 ± 0.220	13.768 ± 0.908	1038.498 ± 11.238	1.493 ± 0.138	0.185 ± 0.005	0.191 ± 0.010	0.188 ± 0.006	276.321 ± 22.487	0.046 ± 0.001
F	BXD74	2	12.761 ± 0.198	15.319 ± 2.199	1007.836 ± 22.749	1.315 ± 0.190	0.188 ± 0.030	0.296 ± 0.042	0.167 ± 0.013	243.388 ± 10.576	0.040 ± 0.005
M	BXD74	4	13.904 ± 0.255	20.768 ± 2.175	1028.833 ± 12.624	1.116 ± 0.112	0.210 ± 0.010	0.353 ± 0.024	0.269 ± 0.026	431.528 ± 41.566	0.048 ± 0.001
F	BXD75	5	12.998 ± 0.340	14.181 ± 1.733	1091.700 ± 3.351	1.645 ± 0.210	0.209 ± 0.012	0.229 ± 0.051	0.108 ± 0.012	166.405 ± 28.776	0.041 ± 0.002
M	BXD75	7	13.382 ± 0.071	15.584 ± 0.843	1075.741 ± 6.332	1.353 ± 0.037	0.240 ± 0.005	0.320 ± 0.022	0.080 ± 0.014	92.476 ± 24.887	0.041 ± 0.002
F	BXD77	5	14.288 ± 0.124	19.224 ± 0.642	1110.150 ± 7.520	1.613 ± 0.158	0.206 ± 0.007	0.278 ± 0.019	0.139 ± 0.007	195.399 ± 15.544	0.045 ± 0.002
M	BXD77	7	14.035 ± 0.107	18.501 ± 0.745	1105.172 ± 12.202	2.071 ± 0.237	0.204 ± 0.010	0.289 ± 0.062	0.173 ± 0.011	287.141 ± 14.822	0.044 ± 0.001
F	BXD78	1	13.776	10.894	1037.972	1.974	0.162	0.164	0.087	133.266	0.038
M	BXD78	3	14.353 ± 0.252	19.580 ± 1.486	1158.264 ± 47.949	1.691 ± 0.341	0.218 ± 0.011	0.416 ± 0.056	0.138 ± 0.028	88.873 ± 39.453	0.057 ± 0.010
F	BXD79	6	12.572 ± 0.534	13.090 ± 1.193	1076.354 ± 20.095	1.361 ± 0.054	0.219 ± 0.011	0.195 ± 0.009	0.078 ± 0.027	116.149 ± 54.555	0.037 ± 0.003
M	BXD79	3	13.752 ± 0.145	18.117 ± 1.248	1086.647 ± 1.851	1.095 ± 0.066	0.266 ± 0.006	0.384 ± 0.045	0.208 ± 0.043	161.983 ± 6.525	0.055 ± 0.005
F	BXD83	4	13.619 ± 0.294	14.743 ± 0.985	1084.406 ± 6.020	1.492 ± 0.131	0.210 ± 0.006	0.278 ± 0.020	0.103 ± 0.005	152.990 ± 6.569	0.041 ± 0.001
M	BXD83	6	13.880 ± 0.278	18.620 ± 1.251	1066.919 ± 6.977	1.678 ± 0.156	0.225 ± 0.007	0.379 ± 0.046	0.175 ± 0.024	255.159 ± 22.043	0.046 ± 0.003
F	BXD84	7	13.262 ± 0.296	12.994 ± 0.753	1044.219 ± 13.135	1.618 ± 0.087	0.194 ± 0.008	0.224 ± 0.011	0.064 ± 0.017	98.203 ± 38.125	0.036 ± 0.002
M	BXD84	1	14.664	18.313	1005.695	1.093	0.199	0.196	0.118	187.898	0.044
F	BXD85	2	12.993 ± 0.087	13.256 ± 0.306	1034.424 ± 5.131	1.628 ± 0.135	0.176 ± 0.012	0.218 ± 0.041	0.096 ± 0.036	138.557 ± 29.270	0.041 ± 0.004
M	BXD85	2	13.654 ± 0.085	16.545 ± 0.951	1054.747 ± 4.393	1.235 ± 0.020	0.214 ± 0.004	0.303 ± 0.031	0.161 ± 0.011	184.167 ± 35.782	0.048 ± 0.003
F	BXD87	2	12.079 ± 0.074	12.133 ± 0.532	1064.210 ± 6.069	1.457 ± 0.080	0.205 ± 0.003	0.228 ± 0.014	0.070 ± 0.001	79.452 ± 4.062	0.042 ± 0.001
M	BXD87	3	12.604 ± 0.293	17.495 ± 1.237	1067.766 ± 12.969	1.183 ± 0.097	0.252 ± 0.005	0.398 ± 0.005	0.166 ± 0.014	124.423 ± 15.673	0.052 ± 0.001
F	BXD89	2	12.973 ± 0.018	13.266 ± 0.040	1062.483 ± 6.841	1.445 ± 0.065	0.205	0.214 ± 0.001	0.064 ± 0.005	87.290 ± 1.713	0.037 ± 0.002
M	BXD89	6	13.092 ± 0.228	14.440 ± 0.567	1048.943 ± 6.637	1.270 ± 0.041	0.230 ± 0.006	0.258 ± 0.020	0.146 ± 0.013	237.926 ± 31.909	0.044 ± 0.002
F	BXD90	9	14.003 ± 0.383	17.074 ± 1.174	1075.584 ± 12.832	1.482 ± 0.100	0.201 ± 0.008	0.363 ± 0.029	0.135 ± 0.015	247.976 ± 27.948	0.038 ± 0.001
M	BXD90	7	14.333 ± 0.221	18.979 ± 0.524	1062.033 ± 9.645	1.432 ± 0.057	0.210 ± 0.010	0.421 ± 0.034	0.170 ± 0.012	357.559 ± 27.835	0.039 ± 0.001
F	BXD95	6	13.343 ± 0.299	16.776 ± 0.927	1037.854 ± 16.495	1.466 ± 0.054	0.206 ± 0.004	0.340 ± 0.025	0.131 ± 0.013	151.017 ± 16.386	0.048 ± 0.002
M	BXD95	4	13.475 ± 0.305	17.151 ± 1.790	1022.122 ± 12.965	1.517 ± 0.132	0.205 ± 0.014	0.388 ± 0.051	0.144 ± 0.031	191.055 ± 41.278	0.044 ± 0.003
F	BXD98	1	14.041	16.740	1074.541	1.211	0.193	0.264	0.148	153.834	0.051
M	BXD98	4	12.930 ± 0.380	13.346 ± 1.141	1048.002 ± 13.947	1.557 ± 0.022	0.194 ± 0.005	0.237 ± 0.013	0.093 ± 0.013	107.846 ± 19.062	0.042 ± 0.001
F	BXD99	3	12.834 ± 0.208	13.892 ± 0.422	1027.939 ± 8.621	1.987 ± 0.155	0.159 ± 0.003	0.188 ± 0.011	0.117 ± 0.017	234.633 ± 33.424	0.038 ± 0.001
M	BXD99	0	N/A	N/A	N/A	N/A	N/A	N/A	N/A	N/A	N/A
F	BXD100	7	13.881 ± 0.283	18.234 ± 1.225	1035.925 ± 13.065	1.318 ± 0.082	0.198 ± 0.005	0.390 ± 0.024	0.150 ± 0.022	203.713 ± 28.279	0.045 ± 0.001
M	BXD100	9	14.321 ± 0.197	21.895 ± 1.044	1068.625 ± 11.070	1.666 ± 0.261	0.229 ± 0.005	0.460 ± 0.029	0.194 ± 0.010	284.210 ± 14.814	0.044 ± 0.001
F	BXD101	3	13.476 ± 0.394	15.290 ± 0.833	1046.598 ± 7.537	1.271 ± 0.071	0.193 ± 0.005	0.226 ± 0.010	0.148 ± 0.006	158.856 ± 5.613	0.047 ± 0.002
M	BXD101	1	13.650	15.891	1026.369	1.398 ± 0.111	0.184 ± 0.009	0.219 ± 0.006	0.154 ± 0.017	224.839 ± 39.199	0.043 ± 0.001
F	BXD102	3	12.677 ± 0.687	14.723 ± 2.329	1032.288 ± 23.284	1.369 ± 0.074	0.188 ± 0.003	0.227 ± 0.017	0.139 ± 0.029	160.763 ± 41.176	0.048 ± 0.002
M	BXD102	8	12.652 ± 0.226	14.952 ± 0.697	1034.898 ± 7.545	1.396 ± 0.093	0.205 ± 0.009	0.313 ± 0.014	0.130 ± 0.018	180.066 ± 20.822	0.045 ± 0.002
	Average per strain	8.67	13.418 ± 0.219	15.948 ± 0.856	1058.565 ± 10.376	1.489 ± 0.114	0.206 ± 0.007	0.286 ± 0.025	0.138 ± 0.017	191.835 ± 25.734	0.044 ± 0.002

**Heritability**									
Males and females combined	Femur length (mm)	Femur volume (mm^3^)	Femur BMD (mgHA/cm^3^)	Femur cortical porosity (%)	Femur cortical thickness (mm)	Femur cortical pMOI (mm^4^)	Femur trabecular bone fraction (BV/TV, %)	Femur trabecular connectivity density (1/mm^3^)	Femur trabecular thickness (mm)
Variance of strain averages	0.44	5.89	833.30	0.064	4.72E‐04	0.007	0.002	5333.20	3.19E‐05
Total variance	0.69	10.14	1182.46	0.117	6.57E‐04	0.011	0.004	9087.32	4.37E‐05
Heritability	0.64	0.58	0.70	0.55	0.72	0.65	0.50	0.59	0.73
**Females only**									
Variance of strain averages	0.47	4.91	686.04	0.057	3.14E‐04	0.003	0.002	5132.51	1.99E‐05
Total variance	0.80	7.91	1311.88	0.107	5.06E‐04	0.007	0.002	7197.09	2.98E‐05
Heritability_F	0.59	0.62	0.52	0.53	0.62	0.46	0.69	0.71	0.67
**Males only**									
Variance of strain averages	0.44	5.89	833.46	0.064	4.72E‐04	0.007	0.002	5333.53	3.19E‐05
Total variance	0.56	10.32	1139.06	0.125	6.64E‐04	0.012	0.004	8339.11	5.32E‐05
Heritability_M	0.78	0.56	0.73	0.52	0.71	0.61	0.48	0.64	0.60

Data are expressed as mean ± SE. Femur cortical porosity, thickness, and polar moment of inertia (pMOI) are representative traits of femoral cortical bone; trabecular bone fraction, connectivity density, and thickness are representative traits of trabecular bone. Broad‐sense heritability *(h* ^2^) = variance of strain averages/total phenotypic variance.

### BXD strain effects and heritability

Under carefully controlled laboratory conditions approximately one‐third of trait variance was accounted for by strain as a factor, even after controlling for age and sex. Heritabilities ranged from 0.29 to 0.78 across all traits (Table 2). The range for females was from 0.36 to 0.69 (mean of 0.59 ± 0.02), for males it was as from 0.29 to 0.78 (mean of 0.57 ± 0.01). Given the large sample size (*n* = 63 strains) and the good control over environmental factors, heritabilities were reassuringly accurate, and had coefficients of error that averaged 1.8%. The highest coefficient of error was 4.3%.

### Sex differences

**Figure 3:**
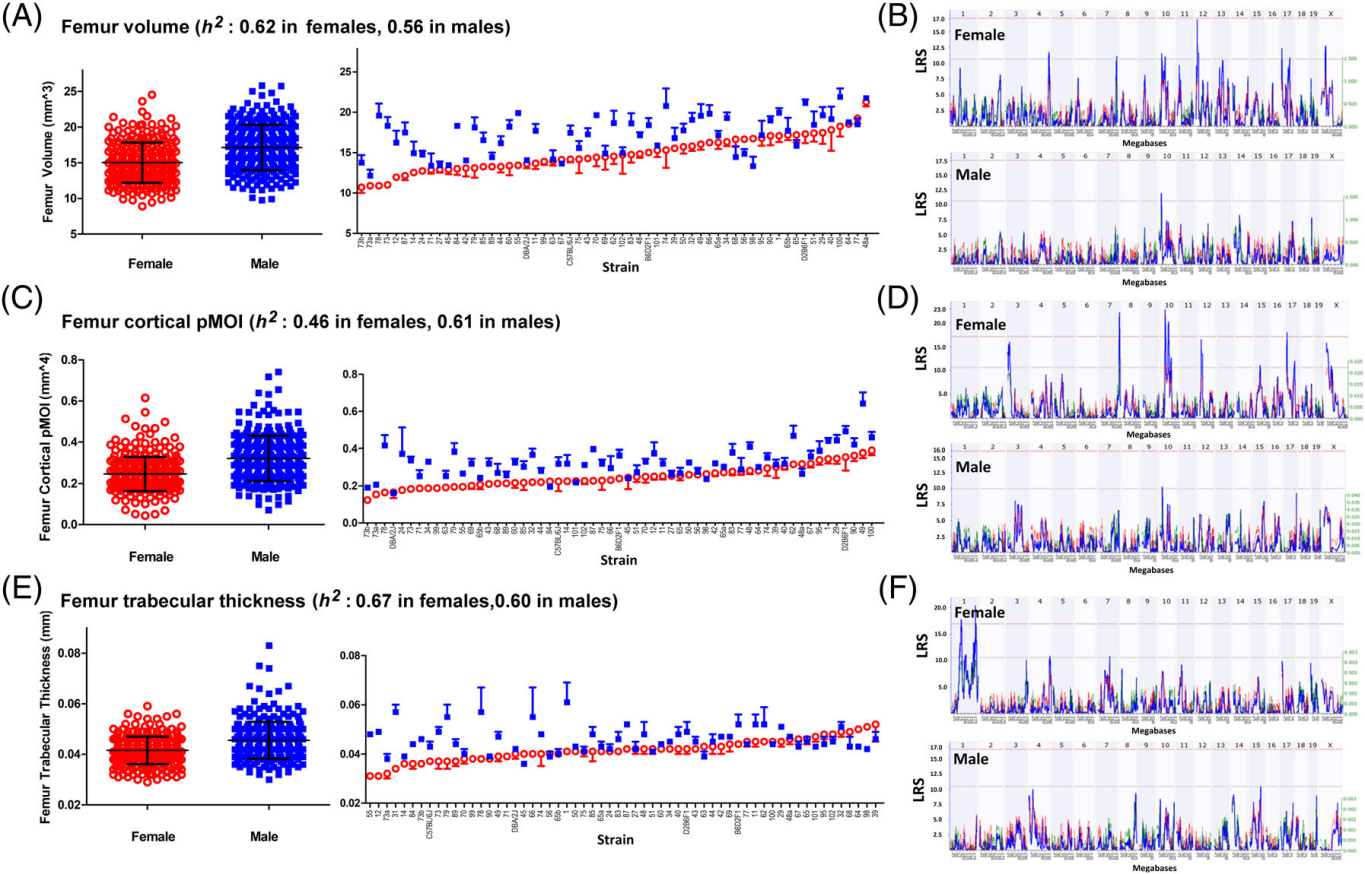
The scatter plots of femur volume (*A*), cortical bone polar moment of inertia (*C*), and trabecular thickness (*E*) of females (red) versus males (blue). Both the mean values of all animals and most strain averages are higher in males. This pattern is consistent in femur volume, cortical polar moment of inertia, and trabecular thickness, and the vast majority of other microtraits. All the corresponding genomic quantitative trait loci (*B*, *D*, *F*) show different locations, peaks, and LRS scores between females and males. Data are expressed as mean ± SEM.

Although heritabilities of male and female traits were closely matched, 0.57 ± 0.01 in males and 0.59 ± 0.02 in females, values for specific bone traits differed significantly, and correlations of heritabilities were surprisingly low (0.29) across 50 common traits. With few exceptions, body weight and absolute values of most traits were greater in males than females (Table 2). For example, body weight, bone length, and bone volume (Fig. 3 *A*, *B*) were all higher in males. Most microtraits also shared this pattern (Fig. 3 *C*–*F*). The few traits that did not show a sex bias were ratio‐based measurements, such as femur material BMD (GN 18182 versus 18232), cortical bone porosity (GN 18185 versus18235), and trabecular degree of anisotropy (GN 18204 versus 18254).

### Correlational statistics of bone traits

Cortical parameters, including cortical volume, thickness, and polar moment of inertia (pMOI), correlated well with each other (Table 3). The correlation between cortical volume (GN 18134) and pMOI (GN 18141) was 0.90, whereas that between cortical thickness (GN 18136) and pMOI was 0.47. These traits also correlated with whole‐bone volume and BMD. For example, the correlation between total femur volume and cortical volume was 0.63 (GN 18131 and 18134), between femur BMD and cortical thickness it was 0.41 (GN 18132 and 18136), and between total femur volume and cortical pMOI it was 0.58 (GN 18131 and 18141). This is not a surprising finding because cortical bone comprises the largest fraction of mouse long bone. In contrast, trabecular bone parameters—bone fraction BV/TV (GN 18146), structure model index (SMI, GN 18148), and trabecular number (GN 18149)—did not correlate well with whole‐bone and cortical bone parameters, except for whole‐bone volume (range from 0.32 to 0.50). Both femoral and tibial traits showed the same pattern. This site‐specificity suggests that the cortical and trabecular components are differentially modulated by gene variants.

**Table 3:**
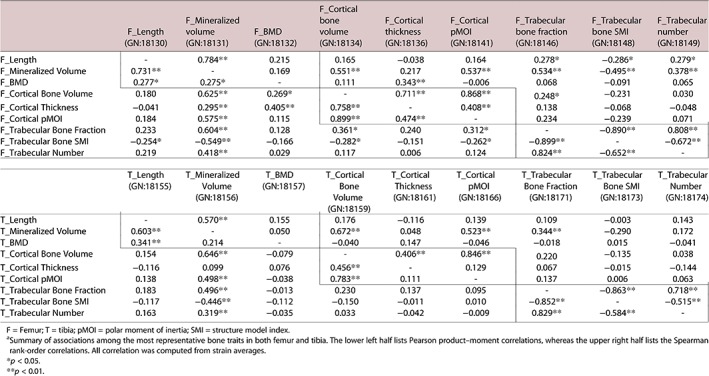
Correlation Matrix for Nine Key Bone Traits in Femur and Tibia^a^

### Genetic correlations of bone traits with gene expression

We used GN to extract the top 1000 transcripts with expression that correlate highly to bone phenotypes. Lists of these top‐ranked transcripts (mean expression level >7.0) were exported to WebGestalt (Web‐based GEne SeT AnaLysis Toolkit: www.webgestalt.org) for GO analysis.^44^, ^45^, ^46^, ^49^ We focused attention on transcripts with highly significant correlations and those that encode extracellular bone matrix, calcium‐modulating molecules, receptors, second messengers, and relevant hormonal agents and cytokines (Supplementary Data S4). For example, femur trabecular fraction (BV/TV) covaries tightly with *Bmp2* and *Bmp7*, as well as with *Ifitm1* and *Ifitm5*. These genes are potential regulators of ossification and bone mineralization.

### Mapping bone microtraits

We generated maps for all phenotypes (GN 18130 to 18279) and identified 16 loci on chromosomes (Chr) 1, 2, 6, 7, 9, 10, 11, 12, 13, 17, and X (Fig. 1 *C*). There were only small differences in QTL peak locations (up to 6 Mb) and linkage statistics using the two genotype files. The average maximum LRS scores for these 16 QTL were 17.2 ± 3.2 using the classic file and 18.2 ± 4.4 using the newer 2017 file. We were surprised to detect no association between heritabilities of traits and the yield of QTL.

We evaluated the impact of age difference on mapping results. We compared mapping results from the complete data set of 597 mice (GN 18130 to 18279, with a log‐age correction) to those from a subset of 484 mice between 65 and 116 days‐of‐age without log‐age correction (GN 18986 to 19086). The Pearson product–moment correlations between full data with age correction and the trimmed data set without age correction ranged from 0.67 to 0.98 (0.93 ± 0.06 SE, *n* = 25 male and 25 female traits).

We performed additional analyses using different mapping algorithms, composite interval mapping, and adjustments for body size (see Materials and Methods section). Seven QTL were highly consistent (Fig. 1 and Supplementary Fig. S1) and associated with bone microtraits of biological interest.^11^ The peak linkage scores for these traits ranged from 17 to 22, and all were genome‐wide significant. These loci accounted for 25% to 35% of strain mean genetic variance (the *r* ^2^ between the best marker in Table 4 and the strain means). Five QTL were related to cortical traits, whereas two were related to trabecular traits, including *Fttf1a* on Chr 1 and *Ttsf11* on Chr 11.

**Table 4:** Robust Quantitative Trait Loci (QTL) for μCT Traits

QTL symbol^a^	Chr	LRS peak location (Mb)	QTL interval (Mb)	Max LRS	Max LRS female vs male	GN ID	Phenotype	Additive effect^b^	Units	Representative SNP	*p* sex by genotype^c^
*Fttf1a*	1	175.68	170.0–185.0	20.5	20.5 / 1.0	18200	Femur trabecular thickness	0.003	mm	rs3682996	0.0305
*Fpmoif7*	7	144.22	141.0–147.5	19.4	19.4 / 6.2	18191; 18192	Femur polar moment of inertia	0.037	g*mm^2^	rs6334210	0.4434
*Fpmoif10a*	10	30.73	30.0–38.0	22.8	22.8 / 1.2	18191; 18192	Femur polar moment of inertia	−0.036	g*mm^2^	rs13480570	0.0287
*Ttnf11*	11	97.97	96.5–101.2	16.8	16.8 / 1.1	18224; 18226	Tibia trabecular number	0.367	1/mm	rs13481180	0.0068
*Fcvf12*	12	15.74	15.0–27.0	19.5	19.5 / 1.1	18189;18183;18184	Femur cortical volume	−0.037	mm^3^	rs3657682	0.1214
*Tctf13*	13	99.63	93.0–102.0	17.8	17.8 / 10.1	18211;18161;18160	Tibia cortical thickness	0.009	mm	rs13481968	0.5527
*Fmoif17*	17	6.04	5.0–9.5	22.3	22.8 / 1.2	18193	Femur moment of inertia around the longer axis	−0.012	g*mm^2^	rs13482851	0.0124

Bold numbers are significant genome‐wide.

a QTL symbol: first letter F or T (femur or tibia), followed by abbreviation of bone phenotype, and f or m (female or male if sex‐specific QTL). The number is the chromosome.

b Negative additive effect means the *B* allele is associated with higher values.

c The sex‐by‐genotype effects *p* values are not corrected for multiple tests.

#### *Sex differences in QTL mapping*

We succeeded in mapping significant QTL for female traits, but surprisingly not for the corresponding traits in males. The seven robust QTL considered above were all detected in females, but most of the corresponding chromosomal regions in males did not even reach the suggestive criterion (Table 4). The only exception was tibia cortical thickness in males (GN 18261), which reached a suggestive level in males (LRS approximately 11). Among the subset of seven most robust QTL, four were associated with sex‐by‐genotype effects. This sex bias in mapping success was unexpected, particularly because heritabilities were so closely matched (Supplementary Data S1). However, we note that prior mouse QTL mapping studies have detected high levels of sexual dimorphism.^50^, ^51^, ^52^, ^53^, ^54^ In addition, male and female body weight covaried well (*r* = 0.69, *n* = 53, GN IDs 18547 and 18548), but QTL also differed, with suggestive peaks on Chr 1 in males (LRS of 15 at 120 Mb and a high *D* allele) and on Chr 8 in females (LRS of 14 at 80 Mb with a high *D* allele). Of importance, QTL for body weight did not match up with any of the bone trait loci. The converse was also true: In comparison with the only QTL detected in males (GN 18248, LRS 19.7), the corresponding female trait (GN 18198) had an LRS of <1.0 at the same location (approximately 35 Mb on Chr 9). Suggestive QTL also shared the strong female bias: 42 in females, 19 in males.

### GO validates known bone‐associated genes and defines new bone‐associated genes

We extracted sets of genes linked to 34 bone‐associated GO terms using femur gene expression data.^17^ For example, the GO‐term bone trabecula formation includes 10 well‐accepted reference genes: *Chad*, *Col1a1*, *Fbn2*, *Grem1*, *Mmp2*, *Msx2*, *Ppargc1b*, *Sfrp1*, *Thbs3*, and *Wnt10b*. We computed the first three principal components and their eigengenes (GN18479, 18480, 18481) that summarize expression variance in this GO reference set. These eigengenes correlate well with femoral traits and with tibial trabecular traits (|*r*| between 0.50 and 0.70). But of much greater interest, these eigengenes can also be used to highlight novel genes that may also be linked to bone biology, more specifically, those that may fall within new QTL.

We extended this analysis to 34 major bone ontology terms, and then computed correlations between eigengenes and the entire transcriptome. We collected the –log*P* values of these correlation coefficients (Supplementary Data S2, see Bone Scores) for thousands of genes. Nearly 200 transcripts (more formally, probes) have average –log*P* bone scores >7.0 across all 34 GO terms. Surprisingly, among the top 20 with very high scores, >9, only three—*Cd276*, *Bmp3*, and *Satb2*—were already included in sets of bone GO genes. Another five—*Col15a1*, *Unc5b*, *Fam78b*, *Dlx6*, and *Nkd*—have been implicated in bone biology, but are not yet part of any bone‐related GO. The remaining 12—*P3h4*, *Unc5b*, *Srpx*, *C1orf198*, *Gxylt2*, *Clec11a*, *Sec31a*, *Bok*, *Kdelr3*, *Prss35*, and *Tmtc2*—all with scores >9 have not, to the best of our knowledge, been linked with bone biology, and are therefore candidates worth more focused analysis and validation. These genes are examples of what we define as a bone ignorome.

**Figure 4:**
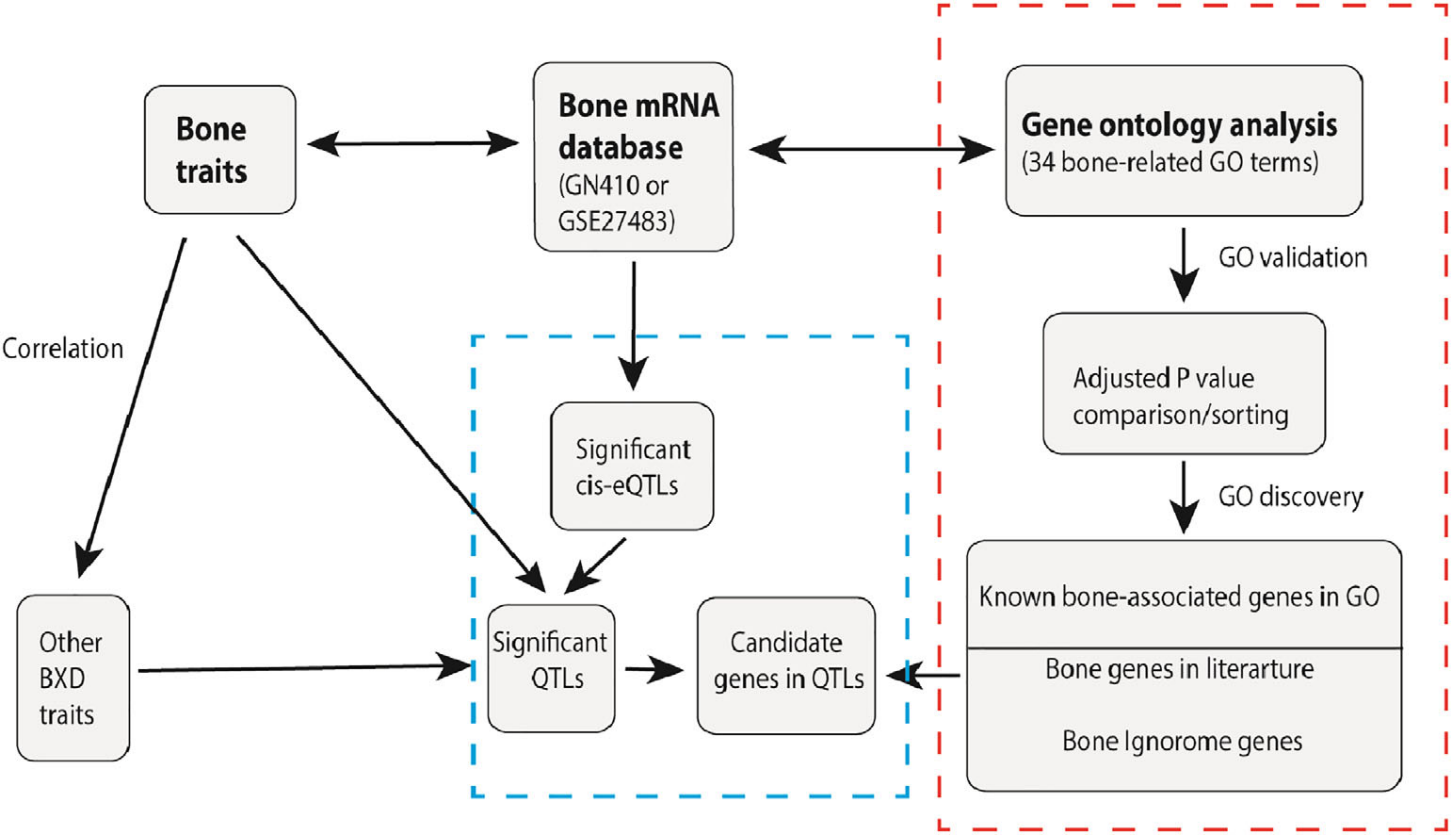
Quantitative trait loci candidate gene analysis workflow. We exploited three primary data sources (bold font at top of figure) for this analysis. Bone traits were correlated to all other BXD traits in GN. Bone traits were also compared against a comprehensive bone mRNA database GN410. The QTL analysis section (box with blue dashed line, bottom center) consists of three boxes that lead to candidate genes in QTL. The gene ontology (GO) analysis section (box with red dashed line to right) summarizes the method used to generate “bone scores” for all probes in the bone mRNA database.

**Figure 5:**
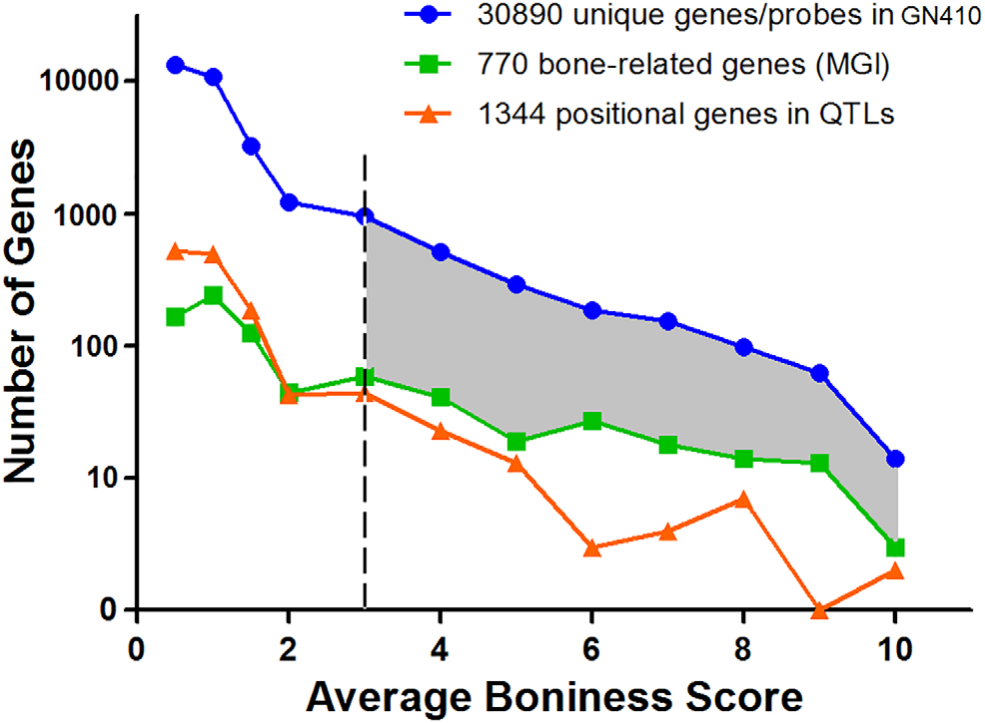
The histogram of average bone scores in three gene sets: (1) Blue line: 30,890 unique protein‐coding genes in femur mRNA (GN410 data set with 46,621 Illumina probes); (2) orange line: 1344 positional genes in 16 quantitative trait loci (QTL; with bone scores from 1638 candidate genes); (3) green line: 770 known bone‐related genes in Mouse Genome Informatics (MGI). Gray area represents 2075 protein coding genes without any known roles in skeletal system biology, but with a bone score >3. Note: The numbers of genes on the *y* axis is on a log10 scale.

We have used three gene data sets to define a bone ignorome (Fig. 4), with the goal of systematically highlighting positional candidates involved in the traits we have mapped. The average bone score of all protein‐coding genes (blue line in Fig. 5) and of all 1344 positional candidate genes (yellow line) are generally low, and averages between these two sets are closely matched: 0.85 ± 0.01 and 0.88 ± 0.03, respectively. As expected, 770 genes already linked to bone GO terms had a much higher mean value of 1.74 ± 0.07 (green line). If a stringent threshold of 3 is chosen, then 2075 genes—the gray area between the blue and green lines (Fig. 5)—meet the criterion of being members of the bone ignorome. Not surprisingly, the percentages of genes with bone scores above 3 in these categories are 7.3%, 7.2%, and 25.2%, respectively (Fig. 5). At more stringent levels of 6 and 9, the numbers of genes above the criterion are 437 and 59, respectively.

#### *Candidate genes*

We defined 212 genes with scores >4 (Supplementary Data S3). Among these, a few had links to bone biology, such as *Ihh*, a number of interferon‐activated genes, and genes in the WNT and ADAM families (Supplementary Data S2). Fifty of the most promising candidate genes with high scores between 5 and 8 were within the seven most robust QTL (Table 5); they can be grouped into three major categories: (1) those genes known to be associated with bone biology and that have been experimentally validated in animal models (*Adamts4*, *Adam12*, *Adam17*, *Ddr2*, *Darc*, *Fkbp10*, *E2f6*, *Ifi204*, and *Grem2*); (2) candidate genes with putative bone functions reported in human studies, but not validated in animal studies (*Ifi202b* and *Greb1*); and (3) bone ignorome genes with high summary scores, but no known links to bone biology (including *Ly9*, *Ifi205*, *Arhgap30*, *Slamf9*, *Ifi203*, *Sde2*, *Usp21*, *Klhdc9*, *Slamf7*, *Cd84*, *Ncstn*, *Copa*, *Tmem63a*, *Ephx1*, *Cd244*, *Atp1a4*, *Slamf8*, *Pyhin1*, *Rgs7*, *Mgmt*, *Frk*, *Krt10*, *Tubg2*, *Krt12*, *Stat5a*, *Trib2*, *Lpin1*, *Pqlc3*, *Hpcal1*, *F2rl1*, *Iqgap2*, *Mrps27*, *Naip5*, *Cmya5*, *Arsb*, *Polk*, *Rgnef*, *Mtap1b*, and *Fndc*).

**Table 5:** Strongest Candidate Genes for Seven Best Quantitative Trait Loci (QTL)

Chromosome	Candidate bone gene validated in animal models: *Symbol* (PMID)	Candidate gene from human studies only: *Symbol* (PMID)	Bone ignorome gene: *Symbol*	Gene description	Cis‐regulation score^a^	Grading score from average^b^	Grading score from max^c^	SNP score^d^	Summary candidate score
1			*Ly9*	Lymphocyte antigen 9	3	2	0	3	8
1			*Ifi205*	Interferon‐activated gene 205	3	2	0	3	8
1	*Adamts4* (22432033)			A disintegrin‐like and metallopeptidase (reprolysin type) with thrombospondin type 1 motif 4	2	2	0	3	7
1			*Arhgap30*	Rho GTPase‐activating protein 30	2	2	0	3	7
1			*Slamf9*	SLAM family member 9	3	2	0	2	7
1			*Ifi203*	Interferon‐activated gene 203	3	2	0	2	7
1			*Sde2*	SDE2 telomere maintenance homolog	3	1	0	3	7
1			*Usp21*	Ubiquitin‐specific peptidase 21	3	1	0	2	6
1			*Klhdc9*	Kelch domain containing 9	3	1	0	2	6
1			*Slamf7*	SLAM family member 7	2	2	0	2	6
1			*Cd84*	CD84 antigen	3	2	0	1	6
1			*Ncstn*	Nicastrin	3	1	0	2	6
1			*Copa*	Coatomer protein complex subunit alpha	2	2	0	2	6
1		*Ifi202b* (18791844)		Interferon‐activated gene 202B	2	1	0	3	6
1			*Tmem63a*	Transmembrane protein 63a	2	2	0	2	6
1			*Ephx1*	Epoxide hydrolase 1, microsomal	2	2	0	2	6
1	*Ddr2* (25805889)			Discoidin domain receptor family, member 2	1	3	0		4
1			*Cd244*	CD244 natural killer cell receptor 2B4	1	1	0	3	5
1			*Atp1a4*	ATPase, Na+/K+ transporting, alpha 4 polypeptide	2	1	0	2	5
1			*Slamf8*	SLAM family member 8	1	2	0	2	5
1	*Darc* (24146983)			Duffy blood group, chemokine receptor	1	1	0	3	5
1			*Pyhin1*	Pyrin and HIN domain family, member 1	2	0	0	3	5
1		*Ifi204* (18287524)		Interferon‐activated gene 204	3	2	0		5
1		*Grem2* (23437003, 23902946)		Gremlin 2, DAN family BMP antagonist	1	1	0	3	5
1			*Rgs7*	Regulator of G protein signaling 7	2	3	0		5
7			*Mgmt*	O‐6‐methylguanine‐DNA methyltransferase	3	2	0	2	7
7	*Adam12* (16869727)			A disintegrin and metallopeptidase domain 12 (meltrin alpha)	1	3	0	1	5
10			*Frk*	fyn‐related kinase	0	3	0		3
11			*Krt10*	Keratin 10	1	3	0	2	6
11			*Tubg2*	Tubulin, gamma 2	2	3	0		5
11			*Krt12*	Keratin 12	1	2	0	2	5
11	*Fkbp10* (26538303, 24777781)			FK506‐binding protein 10	1	3	0		4
11			*Stat5a*	Signal transducer and activator of transcription 5A	3	2	0		5
12	*E2f6* (18366140)			E2F transcription factor 6	3	2	0	2	7
12		*Greb1* (28293781)		Gene regulated by estrogen in breast cancer protein	1	2	0	3	6
12	*Adam17* (22876197)			A disintegrin and metallopeptidase domain 17	3	2	0	1	6
12			*Trib2*	Tribbles homolog 2	1	3	0		4
12			*Lpin1*	Lipin 1	2	1	0	2	5
12			*Pqlc3*	PQ loop repeat containing	2	2	0	1	5
12			*Hpcal1*	Hippocalcin‐like 1	3	2	0		5
13			*F2rl1*	Coagulation factor II (thrombin) receptor‐like 1	3	2	0	2	7
13			*Iqgap2*	IQ motif containing GTPase activating protein 2	2	2	0	3	7
13			*Mrps27*	Mitochondrial ribosomal protein S27	3	1	0	2	6
13			*Naip5*	NLR family, apoptosis inhibitory protein 5	1	2	0	3	6
13			*Cmya5*	Cardiomyopathy‐associated 5	1	1	0	3	5
13			*Arsb*	Arylsulfatase B	2	2	0	1	5
13			*Polk*	Polymerase (DNA directed), kappa	1	1	0	3	5
13			*Rgnef*	Rho‐guanine nucleotide exchange factor	1	3	0	1	5
13			*Mtap1b*	Microtubule‐associated protein 1 B	1	2	0	2	5
17			*Fndc1*	Fibronectin type 3 domain containing 1	1	1	0	3	5

a Cis‐regulation score: = 3 if in bone; = 2 if in cartilage and/or muscle; = 1 if in other tissue.

b Grading score from average bone score: = 1 if average bone score <1; = 2 if between 1 and 3; =3 if >3.

c Grading score from highest bone score: = 1 if highest bone score is between 5 and 10; = 2 if >10.

d SNP score (coding DNA variants score): = 1 if Grantham score <100, = 2 if between 100 and 300; = 3 if >300, or nonsense, frame shift, splice site mutation.

### Combined analysis of mouse candidate genes with QTL and GWAS hits in rat and human

At the level of candidate genes, only 12 out of 212 overlap those already curated in the RGD (Supplementary Data S3, Sheet 1638_sorted_212candidates_ > 4). Ten of these—*Cadm1*, *E2f6*, *Fancc*, *Fcer1g*, *Fkbp10*, *Gja1*, *Hc*, *Ihh*, *Ptch1*, and *Smarcal1*—are defined as having a role in bone biology in mice, one in rats (*Cadm1*), and two in humans (*Grem2* and *Ihh*). RGD lists 295 bone QTL and 646 bone‐associated candidate genes in mouse; 213 QTL and 269 genes in rat; and 70 QTL and 230 candidate genes in human. Collectively, in mouse roughly half of all chromosomes are covered by one or more of 295 QTL. We compared 16 of our prime QTL with those previously mapped in mouse. Fourteen are novel. Three define entirely new loci (labeled in red in Fig. 1 *C*): Chr 6 (*Ttda6*) and Chr X (*FcvfXa* and *FcvfXb*). The other 11 overlap previously reported QTL, but all are μCT‐derived structural traits not directly related to BMD; we therefore consider them novel (labeled in green in Fig. 1 *C*), including *Fttf1a*, *Tcv2*, *Fpmoif7*, *Ftsmoim9*, *Ttdaf9*, *Fpmoif10b*, *Ttsf11*, *Fcvf12*, *Tmoif13*, *Tct13*, and *Fmoif17*. The only two QTL that have similar phenotypes and map positions, and that are therefore provisionally replicated are *Fttf1b* 55 and *Fpmoif10a* 56 (labeled in black in Fig. 1 *C*). Finally, eight of our candidate genes are known to cause abnormal skeletal morphology when knocked out in mice: *Greb1*, *Praf2*, *Timp1*), *Nr1d1*, *Thra*, *Tmem63a*, *Nsun2*, and *Sdhc* had summary candidate scores >4.

Finally, we extended the analysis of candidates by aligning to human GWAS results. Eight of 12 candidates that we tested had relevant human hits (see Supplementary Data S3, Sheet 1638_sorted_212candidates_ > 4). Here we highlight just two. (1) *Grem2* is located on Chr 1 in both mouse and human and overlaps an association for BMD on Chr 1 spanning from 240.1 to 241.1 Mb.^5^ *Grem2* is a member of a family of bone morphogenic protein antagonists expressed in bone that has been linked to low BMD.^57^, ^58^ (2) *Cadm1* is a cell adhesion molecule downregulated in many cancers.^59^ *Cadm1* in human overlaps an association for estimated BMD. In the context of osteosarcoma, *Cadm1* is expressed on the osteoblast cell surface and is used as a marker of differentiation.^60^ Additionally, *Cadm1* plays a role in *NFATc1* modulation of osteoclast activity.^61^

## Discussion

### *Synopsis*

We used high‐resolution μCT imaging to measure bone traits in both sexes of a large cohort of highly diverse strains of mice. We selected a subset of 25 traits from trabecular and cortical compartments of tibia and femur for genetic dissection in each sex across 50 to 61 members of the BXD family. These microstructural traits have heritabilities that range from 30% to 78% in both sexes. We successfully mapped 16 QTL—10 for femur and 6 for tibia—and we generated a list of 1638 candidate genes within 1.5 LOD CIs. Surprisingly, no QTL were shared between sexes, and we were far more successful in defining QTL for females than males, suggesting strong sex hormone and reproductive differences in bone genetic architecture^62^ and in the modulation of bone microstructure. For these 16 QTL, we filtered and extracted the seven most consistent loci that we regard to be of highest interest. We nominated 50 genes with strong associations to skeletal homeostasis and that had high summary bone scores using a novel transcriptome–GO annotation strategy. Of this list, seven candidate genes have been linked with bone biology or abnormalities in human and animal models, including *Adamts4*, *Ddr2*, *Darc*, *Adam12*, *Fkbp10*, *E2f6*, and *Adam17*, whereas another four have been linked either in human (*Grem2* and *Greb1*) or *in vitro* animal models (*Ifi204* and *Ifi202b)*. All are worth additional genetic and molecular studies to test their roles in bone biology and their expression patterns in osteoblasts and osteoclasts. Molecular and cellular functions of the remaining 39 genes are still largely unknown, particularly with respect to the skeletal system. Some have remarkably high bone scores and are therefore primary candidates, especially *Ly9*, *Ifi205*, *Mgmt*, *F2rl1*, and *Iqgap2*.

### The significance of computing bone ignorome scores for candidate gene ranking

We compared all 16 bone QTL with hundreds of rodent bone loci listed in the RGD.^63^, ^64^ Of these 16, 3 are completely novel: 1 on Chr 6 (*Tcv2*) and 2 loci on Chr X (*FcvX* and *FcvfX*). The other 13 overlapped known BMD loci, but only two actually had similar phenotypes and map positions: *Fttf1b* and *Fpmoif10a*. The other 10 were specifically linked to μCT bone traits and were therefore novel.

Given the large number of QTL and positional candidate genes we uncovered, we needed to develop efficient and objective methods to evaluate candidates and their potential role in bone biology. Of 1638 genes overlapping locations of QTL, only 36 (approximately 2%) have been linked to any major bone and skeletal system GO term (see Materials and Methods section). Major skeletal system GO terms currently only include approximately 360 of 24,495 genes in the mouse genome (approximately 1.5% of all coding and noncoding genes), and approximately 404 genes when the list is expanded to include rat and human genomes. RGD lists 652 genes associated with bone structure and function in mouse: roughly 2.7% of all genes. Even this higher value is likely to seriously underestimate the number of genes and the fraction of genome associated with the development, structure, function, and homeostasis of bone and the skeletal system.

An innovation of the present study is that we define a bone ignorome by computing a score using reference gene sets and patterns of gene coexpression. The mean score of well‐known reference genes is 1.74. In comparison, many genes that currently have no known association with bone biology have much higher scores. We applied a reasonably stringent criterion and define genes with a score of 3.0 or higher as members of the bone ignorome. This approach generated a set of 2075 genes potentially associated with bone biology and the skeletal system (Fig. 5)—a value that we believe is more in line with the numbers of genes likely to have an important and possibly selective effect in bone biology.^2^

### Candidate gene ranking

We reviewed 16 QTL to evaluate their replicability when using subsets of data with a narrow age range (65 to 116 days) without age correction (see traits GN 18986 to 19086). This reduced sample size by a third, but did not affect number of strains. As expected, mean linkage scores were reduced by the smaller sample size. Seven loci were insensitive to age as a confounder, and from these seven we nominated 50 candidate genes (Table 5, selecting only those with high summary scores. Two of these are described below as examples of compelling candidates; however, this entire set is worth a further systematic analysis.

*Grem2* (gremlin 2, DAN family BMP antagonist) encodes a member of the BMP antagonist family and is a strong candidate for femur trabecular thickness at the *Fttf1a* locus on Chr 1. The effect of this gene on BMP signaling and osteoblast differentiation has been confirmed in *in vitro* studies.^65^, ^66^ In humans, genetic variants near *Grem2* influence expression in osteoblasts and are associated with fracture risk.^5^ Another study has reported that the minor allele of rs4454537 in *Grem2* is associated with low BMD in the hips of a southern Chinese population.^57^ Our findings suggest that the BXD family, females in particular, would be a good starting point to test genetic and molecular control of *Grem2* and its possible modulation of trabecular thickness.

*Greb1* is a robust candidate for cortical bone volume at the *Fcvf12* locus on Chr 12. This locus also has a strong sex bias with an LRS of 19.5 in females, but only 1.1 in males. *Greb1* is responsive to estrogen in breast tissue.^67^ It is expressed in prostate, and its promoter contains potential androgen receptor‐binding sites.^68^, ^69^ In humans, *Greb1* is associated with BMD at two sites with high fracture rates: the femoral neck and lumbar spine.^70^ However, the association of *Greb1* with bone biology has not been reported previously in animal models. Because both estrogen and androgen are strong modulators of bone remodeling, it is plausible that *Greb1* is a target for both further osteoporotic research and a target for the prevention and treatment of postmenopausal osteoporosis.

In addition to these two obviously strong candidates that already have been characterized in humans, the following genes that have both missense mutations and strong cis eQTL should be the focus of further molecular, experimental, and genetic studies: *Mgmt*, *Mrps27*, *Fndc1*, and *Krt10*. These genes are candidates for femur polar moment of inertia, tibia cortical thickness, femur moment of inertia around the longer axis, and tibia trabecular number, respectively.

### Sex differences

Phenotypic differences of bone traits between sexes were large, but perhaps not surprising. Most values for males were greater than those for females. However, we were surprised by the marked sex imbalance in the yield of QTL. Although heritability of traits was roughly matched—means of 0.59 for females and 0.57 for males—the sex correlations across many traits and strains were at best modest, about 0.43 ± 0.02 SE. Males and females were often littermates, and all phases of phenotyping were carried out without batch processing by sex. Heritabilities did not predict whether a QTL was detected in one sex or the other, nor was the failure to detect QTL in males an artifact of the thresholds we used to declare mapping victory. For example, femur trabecular thickness in females was linked to two strong and independent QTL on Chr 1 with LRS scores of 16 and 20. This trait in males did not even reach a suggestive level anywhere in the genome, and had a peak LRS of merely 7 on Chr 1. We therefore believe that sex differences in mapping reflect underlying differences in genetic architecture and, of course, life history and reproductive roles. Traits in males may be controlled by larger numbers of loci with smaller effects or controlled to a much greater degree by undetected epistatic interactions. Traits in females are likely to be strongly coupled with reproduction and life history. Although sex‐specific modulation of bone and many other traits in animal models and in human studies is accepted almost as a truism,^54^, ^71^, ^72^ studies by Randall and colleagues^73^ and Yang and colleagues^74^ highlight the comparative rarity of sex‐specific gene effects on stature and on BMI. In humans, only the waist:hip ratio is arguably dimorphic, and all seven replicable and significant loci listed by Randall and colleagues for this trait are detected strongly only in females (their Table 1 ^(73)^).

### Site specificity

Over the past decade, more than 150 loci for bone‐associated traits have been mapped in many mouse crosses.^75^, ^76^ Causal gene variants have been successfully defined for more than 10 of these, including *Asxl1*, *Bbx*, *Cadm1*, *Cdh11*, *Fam73B*, *Prpsap2*, *Setdb1*, *Slc38a10*, *Spns2*, *Trim45*, and *Trpsl*.^77^, ^78^, ^79^ Most previous work ‘over 120 of these loci’ has involved only BMD, either of the whole body or bone compartments. In comparison to this simple composite measure, we have focused almost exclusively on μCT traits from deep phenotyping. But for reference to previous work, we have also included BMD measurements. Although BMD by DEXA is still the preferred way to screen and diagnose osteoporosis, it is an aggregate of both cortical and trabecular compartments. We measured three μCT‐derived BMDs from whole bone, cortex, and trabecula in approximately 600 cases, but failed to map any associated loci. In contrast, we were able to detect loci for cortical and trabecular traits, particularly in females.

Remodeling and turnover of cortical and trabecular bones are differentially controlled and regulated.^80^ Trabecular bone is composed of internal rods and plates, forming a lattice that is the primary repository of bone marrow. Because of its close proximity with marrow and marrow‐derived cells, trabecular bone has a higher level of turnover than cortical bone.^81^ Our genetic dissection of these two compartments confirms the distinction. We performed a correlation test, and representative trabecular parameters did not correlate with cortical bone or whole‐bone parameters: bone fraction BV/TV, SMI, or trabecular number. However, individual trabecular and cortical sites did map well, suggesting that gene variants have relatively precise effects on specific regions and compartments.

There was no overlap among the six loci for trabecular bone and the 10 loci for cortical bone. Five of six trabecular loci had significant *p* values for sex‐by‐genotype interactions. In contrast, only two of the femur cortical loci had sex‐by‐genotype effects. Taken together, this confirms that trabecular and cortical microtraits are differentially and independently modulated. These findings underscore the importance of precision phenotyping in mapping and in experimental precision medicine.^82^, ^83^

### Advantages and limitations

This is one of the first genetic studies of bone microarchitecture in mouse using μCT. Our method provides a precise way to quantify and image microarchitecture in trabecular and cortical bone compartments with a resolution of 10 microns or less. One challenge of μCT is the large number of summary values generated per bone. We chose to evaluate and map 25 traits for each bone that are generally regarded of great biological interest. This was still a large number, and raised the issue of the study‐wide false–positive rates. As usual, all mapping had been corrected for genome‐wide testing, but not corrected for numbers of traits—a total of 150 entered into GN. As a result, some loci were likely to be false discoveries. This was the main motivation for extracting only a core set of seven QTL that we regarded as robust in the sense that they are insensitive to variation in age, body weight, genotype file (old versus new), and mapping algorithm (Haley‐Knott versus GEMMA), as well as the distribution and transformation of the phenotype (original data or winsorized data to minimize the impact of outliers). There is inevitably still some risk of false discoveries, but we regard these QTL to be strong enough to warrant independent validation using, for example, CRISPR‐*Cas9* engineering, pharmacological manipulation, or in‐depth omics analyses. A straightforward alternative at this point would also be to extend the study using an independent panel of BXDs or their diallel progeny. There is an additional set of 80 BXD strains that have not been phenotyped at all.^21^ One could also broaden the analysis to include other intercrosses^84^ and Diversity Outbred stock.^85^

Further limitations of this study should be highlighted. First, we used a relatively modest number of genomes (*n* = 50 to 63) and replicates within strain by sex (*n* = 5). We therefore cannot evaluate strain‐specific sex effects. However, we were able to evaluate overall sex differences for all phenotypes and mapping results. Second, the sample size was still too small to detect epistatic interactions, but with the recent boost in the BXD family size to 150 strains,^21^ we can now detect stronger two‐locus interactions.^86^ Third, by incorporating a global analysis of gene ontologies we were able to efficiently filter gene candidates. The GO terms that we selected are obviously not yet complete, and this leads to some false–negatives—many of which we hope we have highlighted using the bone ignorome scores. Fourth, many variants known to be involved in BMD in some human populations will not be detected in the BXD family, or for that matter, even in other human populations. This is principally because of differences in genetic architecture, frequencies of DNA variants, and key environmental factors. For example, a missense variant in *LRP5* (rs3736228) that strongly influences BMD^9^ has a significant lower minor allele frequency in African populations compared with other populations (https://gnomad.broadinstitute.org/). Is this well‐known human variant influential in the BXD mouse family? To test its role in mouse we used a reverse genetic method.^20^, ^87^ The linkage of *LRP5* to BXD bone phenotypes was modest (–logP of 2.46 with GN18279); for this reason, the murine *Lrp5* gene did not enter our list of candidates. However, the phenome‐wide association analysis (PheWAS) in mouse does illustrate how human candidates can be quickly reviewed using both http://www.genenetwork.org and a new PheWAS tool (www.systems‐genetics.org).

### Future directions

The long‐term direction of this work is to transition from locus to gene to mechanism to potential preclinical therapy. The first step is to achieve high‐quality quantitative measures relevant to bone strength and metabolism and to demonstrate a heritable control of variation. The second step is to demonstrate that single loci can be defined with sufficient precision to nominate strong candidate genes. Our work has reached the end of this second stage. What are the subsequent steps that will most efficiently validate candidates and test them as therapeutic targets? One approach would be to produce a multispecies meta‐analysis of genes implicated in bone function. Those shared genes across species will be the most relevant candidates. Another possibility is to systematically study gene‐by‐environmental interactions; something far more efficiently handled using cohorts such as the BXDs in which identical genometypes in sex‐matched pairs can be exposed to several environments or treatments. In addition, pharmacological and molecular methods are needed to validate candidates and mechanisms. Finally, we need to develop higher throughput ways to test therapeutic interventions starting at early stages and using rigorous quantitative methods: what we call experimental precision medicine. Again, cohorts of isogenic animals for which we have superb baseline data will be an essential resource to achieve this last goal, and to evaluate the impact of treatment as a function of genometype and environment.

## Disclosure

The authors declare that they have no competing interests.

## Supporting information


**Supplementary Fig. S1** Genome‐wide interval mapping plot for the seven robust QTL.

**Supplementary Data S1** Supplement_Data S1_BXD_bone_data_Master_Table.

**Supplementary Data S2** Supplement_Data_S2_BoneScore_GO.

**Supplementary Data S3** Supplement_Data_S3_final_candidate_score.

**Supplementary Data S4** Supplement_Data_S4_Table_correlation.
