# Supplementary figures and images for: Genetic Dissection of Femoral and Tibial Microarchitecture
Source: JBMR Plus. 2019 Nov 11;3(12):e10241. doi: 10.1002/jbm4.10241 (PMC6894729; doi:10.1002/jbm4.10241)

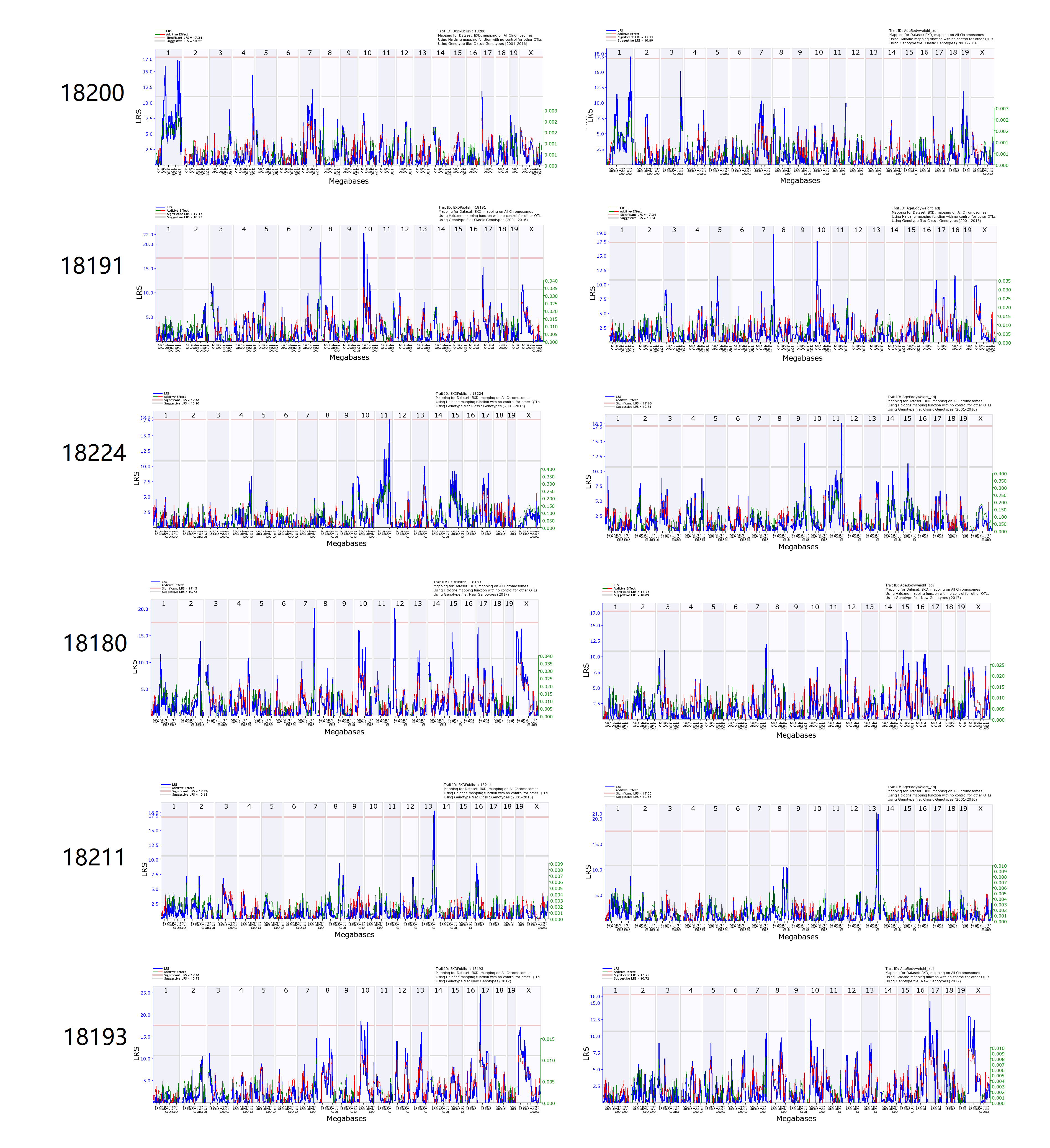

Supplement: Supplementary file 1 — Supplementary Fig. S1 Genome‐wide interval mapping plot for the seven robust QTL. [file JBM4-3-na-s001.png]
